# Functional genomics identifies new synergistic therapies for retinoblastoma

**DOI:** 10.1038/s41388-020-1372-7

**Published:** 2020-06-22

**Authors:** Arthur Aubry, Joel D. Pearson, Katherine Huang, Izhar Livne-bar, Mohammad Ahmad, Madhavan Jagadeesan, Vikas Khetan, Troy Ketela, Kevin R. Brown, Tao Yu, Suying Lu, Jeffrey L. Wrana, Jason Moffat, Rod Bremner

**Affiliations:** 1 Lunenfeld Tanenbaum Research Institute, Mount Sinai Hospital, Sinai Health, Toronto, ON Canada; 2 Department of Laboratory Medicine and Pathobiology, University of Toronto, Toronto, ON Canada; 3 Department of Ophthalmology and Vision Science, University of Toronto, Toronto, ON Canada; 4 Krembil Research Institute, University Health Network, Toronto, ON Canada; 5 Vision Research Foundation, Sankara Nethralaya, Chennai, India; 6 Department of Vitroretina and Ocular Oncology, Sankara Nethralaya, Chennai, India; 7 Donnelly Centre, University of Toronto, Toronto, ON Canada; 8 Department of Molecular Genetics, University of Toronto, Toronto, ON Canada; 9 Present Address: Dualhelix Genetic Diagnostics, Chennai, India

**Keywords:** RNAi, Cancer therapeutic resistance, Chemotherapy, Targeted therapies, Paediatric cancer

## Abstract

Local intravitreal or intra-arterial chemotherapy has improved therapeutic success for the pediatric cancer retinoblastoma (RB), but toxicity remains a major caveat. RB initiates primarily with *RB1* loss or, rarely, *MYCN* amplification, but the critical downstream networks are incompletely understood. We set out to uncover perturbed molecular hubs, identify synergistic drug combinations to target these vulnerabilities, and expose and overcome drug resistance. We applied dynamic transcriptomic analysis to identify network hubs perturbed in RB versus normal fetal retina, and performed in vivo RNAi screens in *RB1*^*null*^ and *RB1*^*wt*^;*MYCN*^*amp*^ orthotopic xenografts to pinpoint essential hubs. We employed in vitro and in vivo studies to validate hits, define mechanism, develop new therapeutic modalities, and understand drug resistance. We identified BRCA1 and RAD51 as essential for RB cell survival. Their oncogenic activity was independent of BRCA1 functions in centrosome, heterochromatin, or ROS regulation, and instead linked to DNA repair. RAD51 depletion or inhibition with the small molecule inhibitor, B02, killed RB cells in a Chk1/Chk2/p53-dependent manner. B02 further synergized with clinically relevant topotecan (TPT) to engage this pathway, activating p53–BAX mediated killing of RB but not human retinal progenitor cells. Paradoxically, a B02/TPT-resistant tumor exhibited more DNA damage than sensitive RB cells. Resistance reflected dominance of the p53–p21 axis, which mediated cell cycle arrest instead of death. Deleting p21 or applying the BCL2/BCL2L1 inhibitor Navitoclax re-engaged the p53–BAX axis, and synergized with B02, TPT or both to override resistance. These data expose new synergistic therapies to trigger p53-induced killing in diverse RB subtypes.

## Introduction

Retinoblastoma (RB) is an aggressive cancer of the infant retina initiated by homozygous *RB1* tumor suppressor gene inactivation or, rarely, by *MYCN* amplification [1–3]. Survival, salvaging the eye and preserving vision depend on disease severity at diagnosis and treatment efficacy. Standardized protocols to prevent tumor spread after intravitreal (IVT) injection have been developed, and improved outcomes have led to adoption of this treatment modality in multiple centers [4, 5]. Intra-arterial chemotherapy has also improved outcome and in advanced cases, alternating this approach with IVT chemotherapy has shown promise without systemic chemotherapy, including for advanced unilateral RB [6, 7]. Notably, combining intra-arterial, IVT and periocular chemotherapy can reduce the time to tumor regression and reduce recurrence in tumors that present with vitreous seeding [8]. Local drug delivery considerably reduces systemic toxicity, however, eye toxicity has been observed with current agents [4, 9]. Thus, innovative therapeutics to improve safety and efficacy are urgently needed. Also, new studies are required to deduce whether *RB1*^*null*^ and “*MYCN*^*amp*^” tumors share similar vulnerabilities.

Precision medicine targets activated oncoproteins, but deleted tumor suppressor genes, such as *RB1*, are not amenable to this approach, and RB tumors exhibit few other mutations [1–3]. It is thus critical to identify key hubs in RB effector networks. MDM2, for example, is expressed in cone precursors, the cell-of-origin of RB, and constrains p53, which is wild type in this cancer [10–12]. Inhibitors targeting MDM2 or its upstream regulators show promise in preclinical studies [13–15]. SKP2, a component of the SCF^SKP2^ ubiquitylating complex, is another promising hub as its loss is synthetic lethal in many *RB1* null contexts, including RB [16]. Indeed, blocking activation of the SCF^SKP2^ complex with the neddylation inhibitor MLN4924 (Pevonedistat) shows promise as a new RB therapy [17]. Such studies illustrate the value in dissecting networks that drive RB cell growth and survival to identify novel therapeutic strategies.

The deployment of RNAi and CRISPR/Cas9 libraries has revolutionized the discovery of cancer drivers and drug resistance mechanisms [18–20]. Genome-wide screens are feasible in vitro, but in vivo studies typically require more focused libraries. To identify high value candidates for in vivo screens, we employed Dynamic Network Modularity (DyNeMo). This tool combines transcriptomic and protein network information to define whether the stoichiometry of co-expressed hubs and partners is altered in cancer vs. normal cells. Previously, DyNeMo pinpointed disrupted hubs influencing outcome in breast cancer [21]. Applying this approach to RB transcriptome data, we identify candidates, establish hits through in vivo RNAi screens in *RB1*^*null*^ and *MYCN*^*amp*^ tumors, and exploit those insights to develop several drug combinations that synergistically kill RB. Moreover, we identify a resistance mechanism and a strategy to resensitize affected RB cells.

## Results

### In vivo screens highlight DNA-repair hubs as drivers in *RB1*^*null*^ and *RB1*^*wt*^*;MYCN*^*amp*^ retinoblastoma

**Fig. 1:**
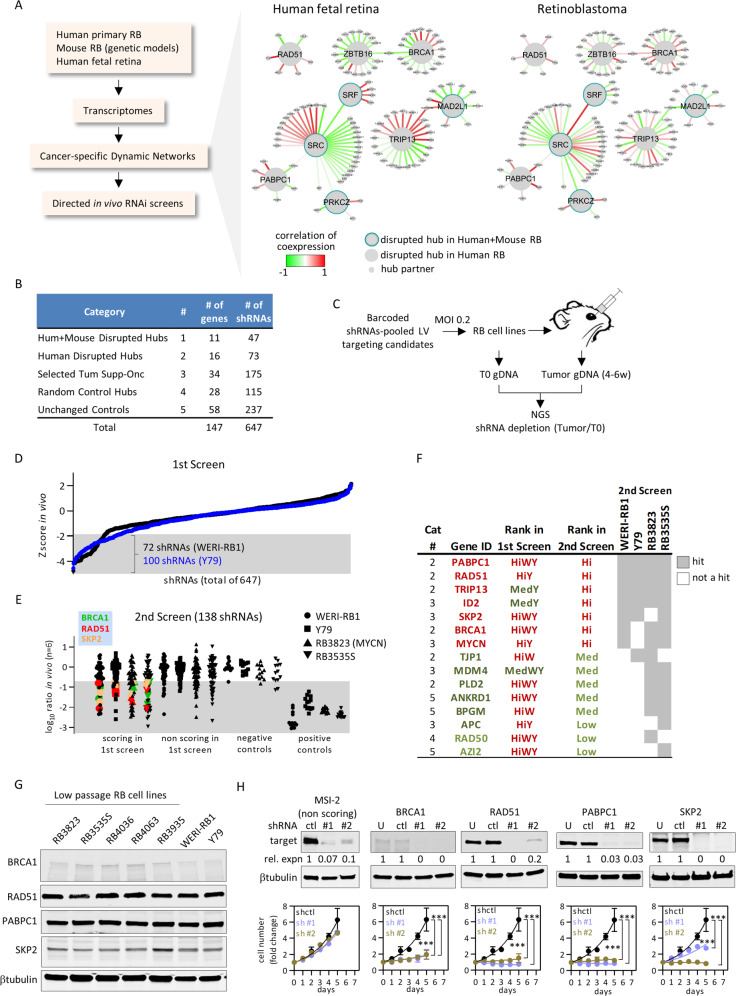
In vivo RNAi screens expose retinoblastoma vulnerabilities. **a** Strategy to identify novel therapeutic targets in RB. Examples of disrupted hub/partners identified by DyNeMo are shown. **b** Sources of the 647 shRNAs targeting 147 genes for the primary in vivo dropout screen. **c** Design of the in vivo shRNA screens. **d** Primary screen data from orthotopic RB xenografts. shRNA enrichment/depletion was determined from *Z*-scores (full dataset in Table S1). Shaded area represents significant dropouts (*Z* < −1.96, *p* < 0.05 two tails). **e** Secondary screen data. In total, 138 shRNAs selected from the primary screen were tested in the indicated four orthotopic RB xenografts. The averaged log ratio tumor/T0 reads (*n* = 6) was plotted. Shaded area represents dropouts (full dataset in Table S1). The recurrent hits BRCA1, RAD51, and SKP2 are highlighted. **f** Summary of hits from the primary and secondary in vivo dropout screens. Hi: high rank; Med: medium rank; W: WERI-RB1; Y: Y79; red: high rank; dark green: medium rank; light green: low rank. **g** Westerns showing expression of the main hits BRCA1, RAD51, PABPC1, SKP2 in multiple RB lines. **h** Screen validation. Y79 cells were transduced with individual shRNAs of the indicated hits or a nonscoring control, knockdown efficiency assessed by western blot, and the effect on growth measured over 5 days by CellTiter-Glo reagent. Data were normalized to d0 and plotted (*n* = 3, mean ± SD, ***p < 0.001 two-way ANOVA, Sidak’s multiple comparisons test).

To select candidates for in vivo shRNA screens we applied DyNeMo [21]. It correlates transcriptional co-expression of hubs (proteins with >4 known partners) and their partners in two conditions (e.g., normal vs. cancer), exposing hubs where these correlations differ. Thus, absolute expression is not relevant but rather the level of network components relative to one another. Using transcriptome data from 21 human *RB1*^*null*^ tumors, and 12 human fetal retinal samples, we identified 27 disrupted hubs (Fig. 1a, b, Fig. S1A, B, Table S1 “DyNeMo result”). Hits were enriched in DNA-repair factors, including BRCA1, RAD51, and XRCC6 (Gene Ontology analysis, *p* = 0.031), and the BRCA1 partner PABPC1. We also assessed orthologous mouse genes from 6 mouse RB models (see “Materials and methods”), which confirmed 11 hubs (Fig. 1a, b, Fig. S1A, B, Table S1 “DyNeMo result”).

We performed in vivo functional shRNA screens with the 27 disrupted hubs and 34 selected tumor suppressors and oncogenes including positive controls known to drive RB (Fig. 1a, b, Table S1 “gene list 1st screen”). We also selected undisrupted hubs and genes with equal expression in tumor vs. human fetal retina (Table S1 “gene list 1st screen”). In total, 647 shRNAs targeting 147 genes were tested. Y79 and WERI-RB1 RB cells were transduced with bar-coded lentiviral shRNAs, drug selected, and genomic (g) DNA collected immediately (time zero (T0)) or from tumors grown from orthotopically transplanted cells (Fig. 1c, and “Materials and methods”). Six tumors per cell line were deep sequenced to identify dropouts (see “Materials and methods”, Fig. S2, Table S1 “1st screen normalized reads”). Of 647 shRNAs, 72 and 100 were significantly depleted in WERI-RB1 and Y79 tumors, respectively (Fig. 1d, *n* = 6, *Z*-score < −1.96, *p* < 0.05). In total, 18 genes had ≥2 significantly depleted shRNAs, of which 9 scored in both lines, while 6 and 3 scored only in Y79 or WERI-RB1, respectively (Fig. 1d, Table S1 “in vivo *Z*-score 1st screen”).

To identify robust and broadly relevant hits, a second iterative in vivo screen was performed with WERI-RB1 and Y79, but also RB3535S, a low passage *RB1*^*null*^ RB cell line, and RB3823, derived from rare *RB1*^*wt*^*;MYCN*^*amp*^ RB [3]. In total, 138 shRNAs/53 genes were processed including: 55 high scoring shRNAs/18 genes from the primary screen; 9 shRNAs/3 borderline genes (defined in Table S1 “2nd screen normalized reads” and “summary table 1st 2nd screen”); negative controls, including 55 shRNA/18 gene non-hits from the first screen, and 4 irrelevant shRNA targets (GFP, RFP, LacZ, luciferase), and; positive controls targeting 12 broadly essential genes [18] (Fig. 1e, Table S1 “2nd screen normalized reads” and “summary table 1st 2nd screen”). The shRNAs were well-represented at T0 as ≥98% yielded ≥ 10 normalized reads in all 4 lines (Fig. S3A). T0 samples were highly correlated, 5/6 WERI-RB1 and 6/6 Y79 tumors formed one cluster, and 6/6 RB3823 and 5/6 RB3535S tumors formed a second cluster (Fig. S3B). Tumor growth was inhibited by 13/13 positive (essential gene) but 0/4 negative control shRNAs, validating the approach (Fig. 1e). 15/18 (83%) hits from the first screen were validated in the second screen, while only 3/18 non-hit controls from the first screen scored in the second (*p* = 0.0002, Fisher’s exact test) (Fig. 1e, Table S1 “ratio tumor vs. T0 2nd screen” and “summary table 1st 2nd screen”). Hits were classified as high, medium, or low quality if they scored in ≥3, 2, or 1 of the 4 cell lines, respectively (Fig. 1f, Table S1 “summary table 1st 2nd screen”). Seven genes were hits in 3/4 or 4/4 lines, indicating good concordance and shared networks across *RB*^*null*^ and *MYCN*^*amp*^ cell lines. These included SKP2 or MYCN (Fig. 1f), confirming prior data [10, 16], and ID2, which drives oncogenesis in other *RB1*^*null*^ and *RB1*^*wt*^*/MYCN*^*amp*^ contexts, including neuroblastoma [22–24]. Paradoxically, ID2 is a tumor suppressor in SV40 large T-driven murine RB [25], so this model does not mimic human RB in this regard. Several highly ranked hits were linked to DNA repair, including RAD51 and BRCA1, which cooperate to mediate homologous recombination (HR) [26], PABPC1, which binds BRCA1 [27], and TRIP13, which promotes DNA repair [28, 29]. All four were hits in both DyNeMo and functional screens, and TRIP13 binds the NHEJ protein XRCC6 (KU80), which DyNeMo flagged as a disrupted hub (Fig. 1a, Fig. S1A, B). We confirmed expression of BRCA1, RAD51, and PABPC1 in multiple low passage RB cell lines, and depletion in Y79 cells, like that of SKP2 but not a negative control locus MSI2, severely inhibited growth in vitro (Fig. 1g, h). Doxycyclin–inducible re-expression of RAD51 further validated the on-target effect of siRAD51 (Fig. S4). Thus, DyNeMo identified relevant targets, exposing overlapping oncogenic networks in *RB1*^*null*^ and *RB1*^*wt*^*/MYCN*^*amp*^ RB, and essential roles for DNA-repair proteins in both tumor subtypes. The retinoblastoma protein (pRB) can promote HR and NHEJ [30–32], *RB1* loss is linked to increased DNA damage [33–36], and the combination of HR gene and *RB1* mutations is beneficial in ovarian cancer [37], but whether directly targeting these repair processes could enhance RB treatment is unclear.

### Sensitivity to BRCA1 independent of non-DNA-repair functions

The pro-tumorigenic role of BRCA1 “tumor suppressor” in RB is intriguing. In addition to DNA repair (see below), there have been claims that it regulates centrosome duplication, heterochromatin integrity and redox [38–40]. In RB cells, BRCA1 foci did not co-localize with pericentrin-positive centrosomes; subcellular fractionation detected BRCA1 in the soluble nuclear compartment but not the cytoplasm where centrosomes are located, and BRCA1 depletion did not alter centrosome number (Fig. S5A–C). BRCA1 loss also did not perturb HP1-positive heterochromatic foci (Fig. S6A, B). Depleting BRCA1 or RAD51 did induce ROS in Y79 cells (Fig. S7A–E). Across four cancer cell lines, increased ROS due to RAD51 depletion correlated with reduced cell numbers, although that was not paralleled in BRCA1-depleted cells (Fig. S7F–H). However, in Y79 or A549 cells, where ROS induction was the greatest, the scavenger trolox reduced ROS, but did not ameliorate G2/M arrest or apoptosis (Fig. S7I–M). Thus, in RB cells BRCA1 does not affect centrosome duplication or heterochromatin stability, and while both BRCA1 and RAD51 suppress ROS, G2/M arrest and cell death induced by their inactivation are ROS independent. Thus, we focussed on DNA damage.

**Fig. 2:**
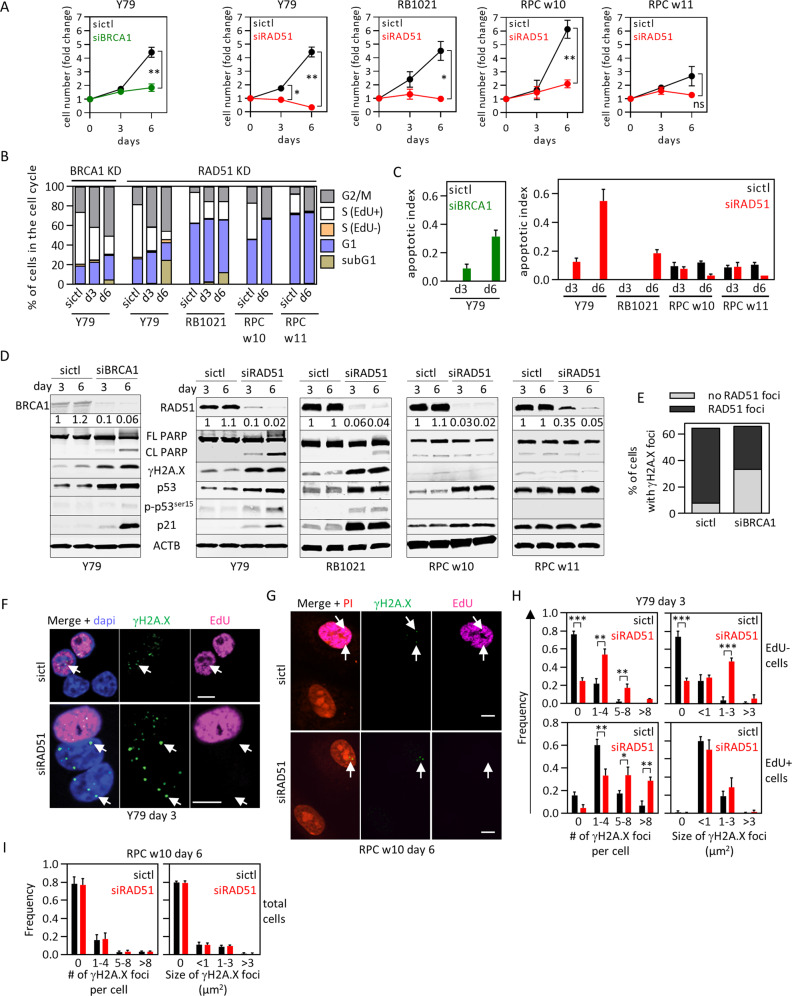
RAD51 loss kills retinoblastoma but not human fetal retinal cells. **a**–**d** The indicated RB tumor cells or RPC were treated with the indicated siRNAs for 6 days, and growth (**a**), cell cycle phase (**b**), apoptosis (**c**) and protein levels (**d**) determined. Representative flow cytometry plots used for (**b**) are shown in Fig. S9A–C. Graph in (**c**) is quantification of PARP cleavage in (**d**) (*n* = 2, mean ± range). **e** Quantification of nuclear RAD51 and γH2A.X foci in Y79 cells treated with siCtl or siBRCA1, detected by immunostaining at day 6 and analyzed by confocal microscopy. **f**–**i** Y79 cells (**f**) or RPC (**g**) were treated with siCtl or siRAD51, labeled with EdU (magenta) and γH2A.X (green) at the indicated timepoints, and confocal images obtained. Arrows indicate γH2A.X foci. The number and size of γH2A.X foci were quantified in EdU^+^ (S-phase) EdU^−^ (non-S-phase) Y79 cells (**h**) or all RPC (**i**). In all cases *n* = 3 (unless specified otherwise). Data in (**a**), (**h**), (**i**) indicate mean ± SD. In (**a**), *p < 0.05, **p < 0.01, ***p < 0.001, ns nonsignificant, two-way ANOVA, Sidak’s multiple comparisons test. In (**h**) and (**i**), *p < 0.05, **p < 0.01, ***p < 0.001 Student *t* test. Scale bars are 10 μm.

Consistent with induction of DNA breaks in *RB1*^*null*^ contexts [33–36], ~60% of untreated Y79 cells had γH2AX foci, predominantly in EdU^+^ S-phase cells (Fig. S8A, B), where BRCA1, RAD51, and γH2AX foci were also detected (Fig. S8A, B). BRCA1 and RAD51 siRNA hindered Y79 cell growth, which correlated with G2/M arrest and apoptosis, evident from subG1 cells, DNA fragmentation, and PARP cleavage (Fig. 2a–d, Fig. S9A, F, G). Depleting RAD51 had a similar effect in another cell line, RB1021 (Fig. 2a–d, Fig. S9B). Micronuclei suggested mitotic stress following DNA damage (Fig. S9D, E). Depleting BRCA1 or RAD51 induced γH2AX, p53 expression, DNA damage-related p53 activation (Ser15 phosphorylation), and the p53-target gene p21 (Fig. 2d). The degree of apoptosis and growth inhibition was slightly less following BRCA1 depletion (Fig. 2a–d, Fig. S9A, F, G), and indeed BRCA1 loss did not completely eliminate RAD51 recruitment to γH2AX foci (Fig. 2e). The latter might reflect remnant BRCA1 and/or proteins that promote HR in BRCA1-deficient cells [29]. We focussed subsequent assays on RAD51. Immunostaining confirmed γH2AX-induction following RAD51 knockdown, an increased number of foci/cell in both S- and non-S-phase cells, and enhanced foci size in the latter (Fig. 2f, h). Thus, persistent DNA damage correlates with the phenotypic effects of BRCA1/RAD51 depletion in RB cells.

### RAD51 depletion kills RB tumor but not human retinal progenitor cells

Sensitivity to RAD51 depletion might be a general feature of dividing fetal retinal cells rather than a specific property of RB. Human RB arises from ectopically dividing cone precursors but normal cones do not divide, so the closest possible normal cell comparison would be retinal progenitor cells (RPC), from which cones are derived. Thus, we cultured RPC from week 10 (w10) and w11 fetuses. RPC-w10 grew similarly to RB cells while RPC-w11 grew more slowly (Fig. 2a). We confirmed that the cells were cycling with Ki67 and cyclin D1, and validated retinal neurogenic progenitor identity with multiple markers (Chx10, Sox2, Neurod1, Pax6); the cells also expressed glutamine synthetase, which is a late progenitor/Muller glia marker, but lacked Gfap ruling out contaminating astrocytes derived from nonretinal cells and implying some differentiation in culture (Fig. S10A–D). Progenitor and Müller glia transcriptomes are 50% identical [41], thus either provides a reasonable baseline for RAD51 dependence in dividing retinal cells. Assessment of γH2AX and RAD51 foci revealed basal levels of damage and HR activation in RPC, which was, as expected, mainly in S-phase, and the proportion of affected cells was somewhat lower in RPC vs. RB cells (Fig. S8A, C). RAD51 levels were similarly depleted by siRNA in RPC and RB cells (Fig. 2d), but despite G2/M arrest in both scenarios, p53 activation and persistence of γH2AX foci, and increases in micronuclei, fragmented DNA, subG1 cells, and PARP cleavage were all unique to RB cells (Figs. 2b–d, 2F–I, Fig. S9A–G). Thus, RAD51 depletion selectively kills RB cells.

### CHK1/2 drive cell cycle arrest and p53-mediated apoptosis in HR-depleted RB cells

**Fig. 3:**
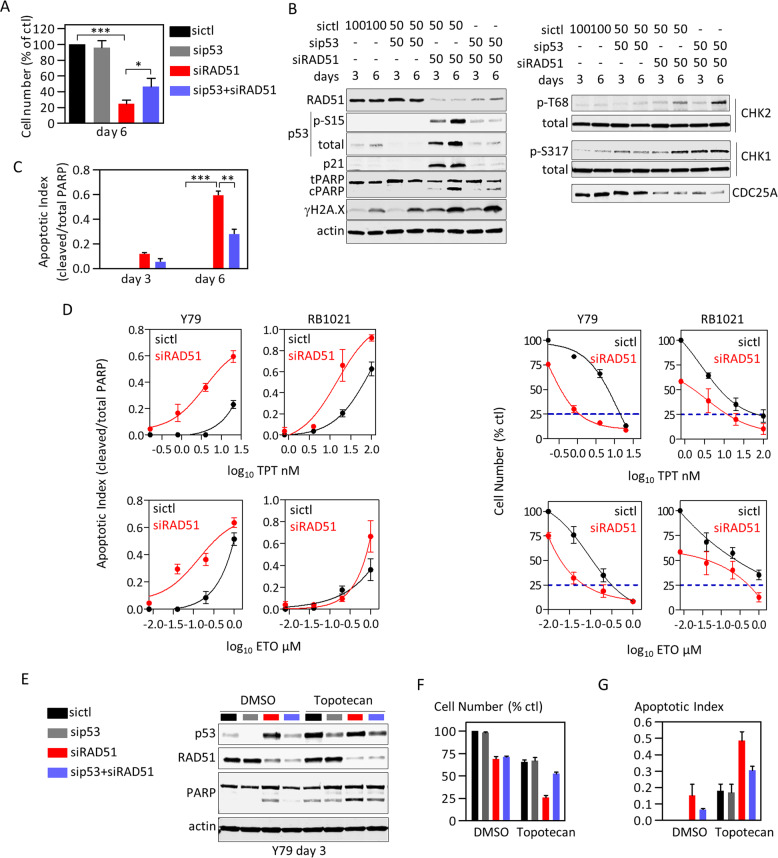
Depleting RAD51 sensitizes RB cells to topoisomerase inhibitors by promoting p53-mediated death. **a**–**c** Y79 cells were treated with the indicated siRNAs for 3 or 6 days (nM indicated in (**b**)) and growth (trypan blue counts, (**a**)), protein levels (westerns, (**b**)), and apoptosis (PARP cleavage, (**c**)) assessed. (*n* = 3, mean ± SD, *p < 0.05, **p < 0.01, ***p < 0.001 ordinary one-way ANOVA, Tukey’s multiple comparisons test). **d** Y79 or RB1021 cells were treated with siCtl or siRAD51 plus either TPT or ETO, and dose-response curves obtained for cell number (trypan blue) or apoptosis (PARP cleavage, representative blot in Fig. S13A). (*n* = 3, mean ± SD). **e**–**g** Y79 cells were treated with siRNAs and drugs as indicated. At day 3, cells were harvested for westerns (**e**), cell counts (**f**), or apoptosis (PARP cleavage, (**g**)) (*n* = 2, mean ± range).

Next, we investigated the molecular basis of HR sensitivity. Above, we showed that RAD51 depletion activated p53, and indeed RB cells survived when both RAD51 and p53 were depleted (Fig. 3a–c). Following DNA damage, ATM/ATR phosphorylate CHK1/2 kinases, which promote degradation of CDC25 phosphatase, causing G2/M arrest [42–44]. Indeed, depleting RAD51 drove phosphorylation of ATM/ATR sites on CHK1 and CHK2 at Ser317 and Thr68, respectively, accompanied by CDC25A downregulation (Fig. 3b). ATM or CHK2 inhibitors blocked p53 and H2AX phosphorylation, preventing PARP cleavage, and while CHK2 inhibition modestly rescued CDCD25A levels, CHK1 inhibitor PF-477736 was more potent (Fig. S11A, B). These data reveal functional relevance for all three kinases in RAD51-depleted RB cells.

Depleting p53 in siRAD51-treated cells did not fully rescue cell number (Fig. 3a). Cell cycle analysis showed that while depleting p53 reduced subG1 cells (confirming PARP analysis), it did not affect G2 arrest (Fig. S11C). CHK1/2 phosphorylate CDC25 proteins after DNA damage [44, 45], and as noted above, CHK inhibitors rescued CDC25A levels, suggesting a possible mechanism for G2 arrest (Fig. S11B). Indeed, treating RAD51-depleted cells with either of two CHK1 inhibitors (PF-477736, MK-8776) or CHK2 inhibitor II reduced G2 cells, and combining MK-8776 with CHK2 inhibitor II potentiated the effect (Fig. S11D). Alone, CHK inhibitors also partially restored the proportion of replicating (EdU^+^) cells and total cell number, and rescue was nearly complete when CHK1/2 inhibitors were combined (Fig. S11E, F). Thus, CHK1/2 cooperatively promote G2 arrest in HR-deficient RB cells, likely through CDC25, and cell death through p53.

### HR-defective RB cells are hypersensitive to topoisomerase inhibitors in a p53-dependent manner

Combining DNA-damaging agents with DNA-repair inhibitors can improve chemotherapeutic efficacy [46, 47]. This approach has yet to be explored for RB management, even though DNA-damaging agents such as topotecan (TPT), etoposide (ETO), and melphalan are used routinely to reduce tumor load [48]. We asked whether inhibiting HR potentiates TPT and/or ETO.

TPT or ETO induced RAD51 foci in 95 or 60% of cells, respectively, and RAD51-depleted cells lacked foci, confirming antibody specificity (Fig. S12A, B). Fifty percent of vehicle-treated cells were RAD51^+^ but with 1–3 foci, whereas 76% of RAD51^+^ TPT-treated cells had >12 foci/cell, and 63% of RAD51^+^ ETO-treated cells had 4–9 foci/cell (Fig. S12A–C). RAD51 foci overlapped γH2A.X in all drug treatments, demonstrating coincident DNA damage and HR (Fig. S12A). Consistent with previous work, TPT and ETO induced potent G2 arrest (Fig. S12D) [49, 50]. High throughput analysis of γH2A.X foci in the cell cycle with imaging flow cytometry (ImageStream®X) confirmed that TPT blocked cells in G2 due to extensive DNA damage in S + G2 (Fig. S12E–I). Thus TPT and ETO induce DNA damage and activate RAD51 in RB cells.

Next, Y79 and RB1021 cells were treated for 72 h with control or RAD51 siRNA (efficiency > 90%, Fig. S13A) plus either DMSO or increasing doses of TPT or ETO. Depleting RAD51 enhanced apoptosis in response to multiple concentrations of either drug in Y79 cells and similarly for TPT in RB1021 cells, although for unclear reasons sensitivity was elevated only at the highest dose of ETO in RB1021 cells (Fig. 3d, Supplementary Fig. S13). In Y79 cells, siRAD51 reduced TPT EC75 16-fold (Control 13.5 nM vs. siRAD51 0.84 nM), or 11-fold for ETO (330 nM vs. 32 nM), and 13-fold or 4-fold in RB1021, respectively (Fig. 3d). PARP cleavage and annexin V staining confirmed increased apoptosis (Fig. 3d, Fig. S13A, B). For example, the apoptotic index with 20 nM TPT in Y79 cells was reached with only 0.8 nM following RAD51 depletion, a 25-fold reduction. Enhanced cell death was p53 dependent (Fig. 3e–g). Thus, disrupting HR sensitizes RB cells to clinically relevant topoisomerase inhibitors.

### A small molecule RAD51 inhibitor synergizes with topo inhibitors to promote apoptosis in RB cells

Thus far, our network analysis and RNAi screens link HR to RB cell survival. Depleting RAD51 selectively kills RB compared to fetal retinal cells, through activation of a CHK1/2-p53 DNA-damage response pathway. Moreover, RAD51 knockdown sensitizes RB cells to clinically relevant chemotherapeutic drugs. Next, therefore, we tested whether a small molecule inhibitor of RAD51 polymerization, B02 [51], could also synergistically kill RB cells. This agent enhances cisplatin, but only modestly enhances ETO and TPT efficacy in breast cancer MDA-MB-231 cells [52]. B02 dose-response curves on three RB lines revealed EC50s of 15–20 μM (Fig. S14A), comparable to other cell lines [52]. In soft agar, B02 impaired Y79 colony formation with an EC50 of 7 μM (Fig. S14B). Cells treated with 25 μM for 48 h lacked RAD51 foci (Fig. S14C). Like RAD51 depletion, B02 caused G2/M arrest, elevated DNA damage, and at higher concentrations (25 μM) induced p53 activation and apoptosis in Y79 cells (Fig. S14D–F). RB cells were twice as sensitive as RPC (EC50 15 vs. 30 µM, Fig. S14A). Cancer selectivity was retained even after long-term exposure (9 days) (Fig. S14G–I). Thus, RAD51 depletion or inhibition is selectively lethal.

**Fig. 4:**
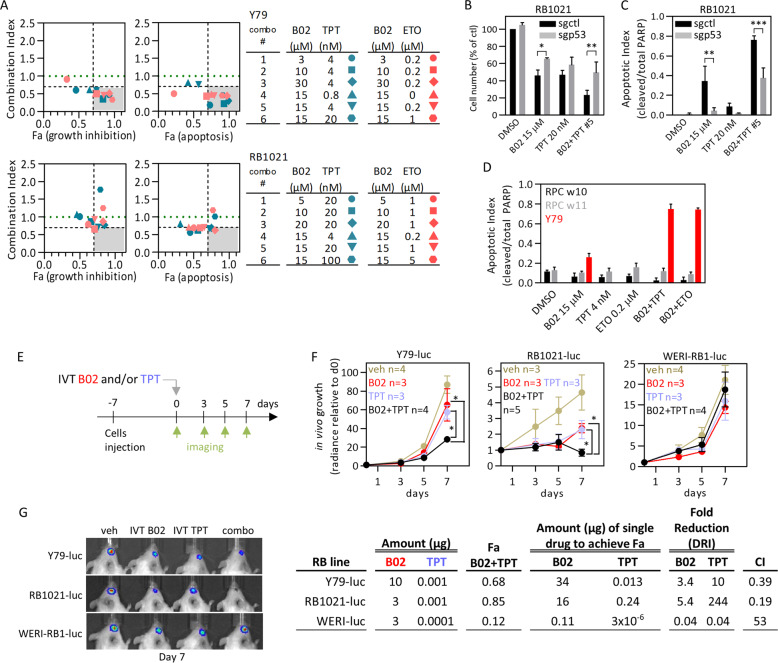
p53-dependent and tumor-selective synergy of B02 with standard RB chemotherapy. **a** Combination Index (CI) vs. effect (Fa) plots for two RB cell lines treated with the indicated two-drug combos of B02, topotecan (TPT), and etoposide (ETO). Turquoise and pink data points show B02 + TPT and B02 + ETO combos, respectively. The gray area delineates potent synergistic combos (CI < 0.7 and Fa > 0.7), and the green dotted line CI = 1; detailed growth inhibition curves, PARP westerns, and quantification curves are shown in Fig. S15A, B. **b**, **c** RB1021 cells were transduced with control or p53 sgRNA lentivirus, selected in puromycin, then drug-treated as indicated. Cell number (trypan blue, (**b**)) and apoptosis (PARP cleavage, (**c**)) were assessed on day 3 (*n* = 3, mean ± SD, *p < 0.05, **p < 0.01, ***p < 0.001 ordinary one-way ANOVA, Tukey’s multiple comparisons test; representative PARP western in Fig. S16A). **d** RPC or Y79 tumor cells were treated with the indicated single drugs or combo #5 from (**a**), and apoptosis quantified on day 3 (PARP cleavage, *n* = 2, mean ± range; representative PARP western in Fig. S16G). **e** Timeline to assess IVT B02 and/or TPT in three orthotopic RB xenograft models. **f** Based on day 7 data from Fig. S17A, B and plotted as dose response in Fig. S17C, subEC50 doses of B02 and TPT were selected (indicated in columns 2 and 3 of the table below the growth curves) and tested alone or together on the three indicated xenograft models (mean ± SD, *p < 0.05 two-way ANOVA, Sidak’s multiple comparisons test). DRI and CI are also indicated on the table. **g** Representative images of the radiance signals for the three RB tumors treated as indicated after 7 days are shown.

B02, like siRAD51, also enhanced TPT and ETO toxicity (Fig. S15A, B). To assess synergy, we determined the Combination index (CI) for growth inhibition or apoptosis (fractions affected (Fa)), then plotted Fa vs. CI for each combo concentration [53]. Several combos were synergistic (CI < 1) in both Y79 and RB1021, for both drug combinations, and some were hypersynergistic (CI < 0.7, Fa > 0.7) (Fig. 4a). Multiple B02 + TPT combos also synergistically impaired 3D colony growth of Y79 cells (Fig. S15C, D). Like siRAD51+TPT (Fig. 3e–g), killing by B02 + TPT was p53-dependent, as CRISPR-mediated p53-deletion rendered RB1021 resistant to B02 alone or B02/TPT (Fig. 4b, c, Fig. S16A). BAX is a well-known proapoptotic p53 target [54], constrained by antiapoptotic proteins such as BCL2L1 (BCL-XL) [55], and the BAX/BCL2L1 ratio was increased by TPT, and more-so the combo, all of which was p53-dependent (Fig. S16A). Moreover, with the same conditions and cutoffs there was no B02 + TPT synergy in p53-mutant MDA-MB-231 cells (Fig. S16B, C). Synergistic death in RB cells was confirmed by increased apoptotic morphology and Annexin V/Fxcycle Violet staining (Fig. S16D, E). In contrast, drug combos did not affect RPC (Fig. 4d, Fig. S16F, G). Thus, B02 recapitulates RAD51 depletion, including cancer-specific p53-dependent synergy with TPT and ETO.

### B02 and TPT synergize in vivo

Next, we assessed in vivo efficacy. It is important to define suboptimal drug doses for synergy studies. A single IVT injection of B02 revealed dose and time dependent responses in three orthotopic RB xenograft models (Fig. S17A–C). By day 7, 30 μg B02 inhibited Y79, RB1021, and WERI-RB1 tumors 72%, 85%, and 50%, respectively, reduced division (EdU, Ki67) and increased apoptosis (Active Caspase 3 (AC3)) without affecting retinal morphology (Fig. S17D, E). Thus, B02 is a potentially novel RB therapeutic. IVT TPT is effective in human vitreal disease [48], but in preclinical models IVT delivery has only been tested on retinal disease [56]. In vivo analysis showed dose-responsiveness in all three RB models without toxicity up to 1 μg (Fig. S17B, C, E). After defining subEC50 doses, we next tested synergy. IVT B02 + TPT inhibited Y79 and RB1021 tumors better than either drug alone (Fig. 4e–g), matching in vitro data. At endpoint (day 7), the combo inhibited Y79 or RB1021 tumor growth 70% or 85% vs. 35% or 50% with single drug, respectively (Fig. 4f, g). Dose reduction index (DRI) compares the amount of single drug required to achieve the effect with the amount used in the combo. The in vivo calculated DRIs for Y79 were 3.4 (10 μg vs. 34 μg) for B02, and 13 (0.001 μg vs. 0.013 μg) for TPT, while for RB1021 they were 5.4 (3 μg vs. 16 μg) for B02 and 244 (0.001 μg vs. 0.24 μg) for TPT (Fig. 4f). CIs were 0.39 in Y79 and 0.19 in RB1021, indicating potent synergy in both RB tumors (Fig. 4f). Unexpectedly, a similar trial with WERI-RB1 revealed a CI > 1, indicating antagonism (Fig. 4f). Thus, HR inhibition potently synergizes with TPT in a subset of RB tumors, offering a strategy to reduce toxicity with IVT delivery [57].

### p53 induction of p21 mediates resistance to B02 and TPT

**Fig. 5:**
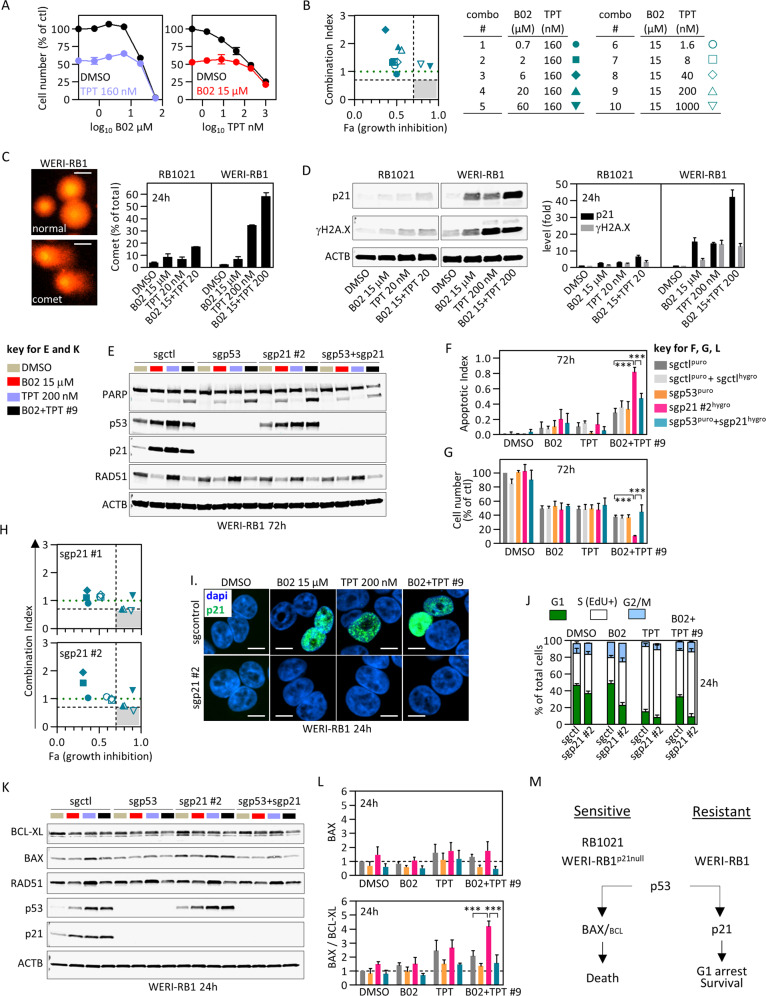
The p53–p21 axis underpins resistance to B02 and TPT. **a** WERI-RB1 cells were treated with increasing concentrations of B02 (left) or TPT (right) together with vehicle (black) or EC50 doses of TPT (blue) or B02 (red) and cell number determined after 3 days (CellTiter-Glo, *n* = 2 ± range). **b** The combos tested in (**a**) are summarized and CI vs. effect (Fa) plotted. Gray area and green line as in Fig. 4a. Sensitive RB1021 or resistant WERI-RB1 cells were treated 24 h with ≈EC50 B02 and/or TPT and DNA double-stranded breaks assessed by alkaline comet assay (**c**) and γH2A.X westerns (**d**). Examples of propidium iodide-stained normal nucleoids, DNA comets, and normalized comet quantification (*n* = 2 ± range) are shown in (**c**). Scale bars are 10 μm. In (**d**), p21 and γH2A.X expression were also quantified (*n* = 2 ± range). **e**–**g** Deletion of p53, p21, or both with CRISPR/Cas9 sgRNA lentiviruses was performed in WERI-RB1, and sensitivity to B02, TPT, and B02 + TPT combo #9 (from (**b**)) was assessed after 3 days by tracking apoptosis (representative blot in (**e**), quantified in (**f**)), and cell number (**g**) (*n* = 3, mean ± SD, ***p < 0.001 ordinary one-way ANOVA, Tukey’s multiple comparisons test). p53, p21, RAD51 levels were also assessed in (**e**). Additional westerns are shown in Fig. S19H. **h** B02/TPT synergy assay in p21-deleted cells, run as for parental cells in (**a**, b). The related growth inhibition curves for the two sgp21 tested are shown in Fig. S19G. **i**, **j** WT or p21-null WERI-RB1 cells were treated 24 h with single drugs or combo #9 (from (**b**)) and p21 localization assessed by immunostaining (**i**), or cell cycle phase defined by EdU and fxcycle staining followed by flow cytometry (**j**) (*n* = 2, mean ± range). Representative flow plots are shown in Fig. S19I. Scale bars are 10 μm. **k** The experiment in (**e**–**g**) was repeated but protein levels were assessed at 24 h. **l** BAX and BCL-XL western blots in (**k**) were quantified, normalized to actin and DMSO, then levels were plotted as indicated (*n* = 3, mean ± SD, ***p < 0.001 ordinary one-way ANOVA, Tukey’s multiple comparisons test). **m** Schematic summarizing the distinct p53 response to B02/TPT in sensitive (left) or resistant (right) contexts.

Drug resistance is a major reason for treatment failure, thus we investigated why WERI-RB1 cells resist B02 + TPT treatment. Resistance was recapitulated in vitro (Fig. 5a, b), providing a setting to test hypotheses. Unique to WERI-RB1, B02 alone or with TPT caused autophagy-like effects, including vacuolization and flattening (Fig. S18A). However, the autophagosome marker LC3-II was not induced, and two autophagy inhibitors did not affect LC3-II nor sensitize cells to B02 and/or TPT (Fig. S18B–D). Vacuoles in B02-treated cells lacked mitochondria, arguing against the idea that mitophagy inhibits the intrinsic apoptotic cascade (Fig. S18E).

Comet assays and γH2A.X westerns revealed that B02 + TPT increases DNA damage vs. single drug, but unexpectedly, the combo induced greater damage in resistant WERI-RB1 vs. sensitive RB1021 cells (Fig. 5c, d). The p53 target, p21 can arrest cells to permit DNA repair [58], and indeed, 24 h post treatment it was induced to higher levels in WERI-RB1 cells relative to RB1021 with either drug alone and maximally with the combo (Fig. 5d). By 72 h p21 was lower in combo vs. single drug-treated cells, reflecting greater apoptosis in the former (Fig. 5e). CRISPR-mediated p21-deletion increased apoptosis and reduced cell number specifically in combo-treated WERI-RB1 cells, thus drug resistance is p21-dependent (Fig. 5e–g, Fig. S19A–F). Dose-response experiments identified drug concentrations that synergistically killed p21-deficient WERI-RB1 cells (Fig. 5h, Fig. S19G), contrasting no synergy in parental cells (Fig. 5a, b). Cytoplasmic p21 inhibits caspase 3 [59], but the protein was nuclear (Fig. 5i). RAD51 levels, which were reduced by B02, were unaffected by p21 loss (Fig. 5e), suggesting another mechanism of resistance. In WT cells, TPT caused S-phase arrest, but adding B02 increased G1 cells to levels approaching vehicle or B02-only treated cells, thus p21 may protect combo-treated cells through G1 arrest (Fig. 5j, Fig. S19I). Indeed, deleting p21 dramatically increased the fraction of combo-treated cells in S-phase (Fig. 5j, Fig. S19I). Thus, while the combo induces greater DNA damage in drug-resistant cells, the counterintuitive reduction in death is due to more robust p21 induction, G1 arrest and protection of a subset of cells.

### Re-engaging the p53–BAX axis to kill B02/TPT-resistant cells

The p53–BAX axis underpins B02 + TPT synergy in RB1021 cells (Fig. 4b, c, Fig. S16A). p21 is also a p53 target, thus conceivably p53 protects WERI-RB1 cells because p53–p21 predominates over p53–BAX signaling. Indeed, 24 h after B02 + TPT treatment, the BAX/BCL2 ratio was markedly induced in p21-deleted WERI-RB1 compared to resistant parental cells (Fig. 5k, l). p53 was essential for the induction of p21 in WT WERI-RB1 cells (Fig. 5k, l). In line with a context-dependent role, deleting p53 alone in WERI-RB1 cells did not alter drug responsiveness because neither p21 nor BAX/BCL-XL was induced (Fig. 5e–g). Thus, p53 promotes or antagonizes B02 + TPT synergy in RB cells depending on whether the BAX or p21 pathways predominate, and removing p21 restores BAX dominance in resistant cells (Fig. 5m).

### Synergism with Navitoclax overcomes resistance to B02 + TPT

**Fig. 6:**
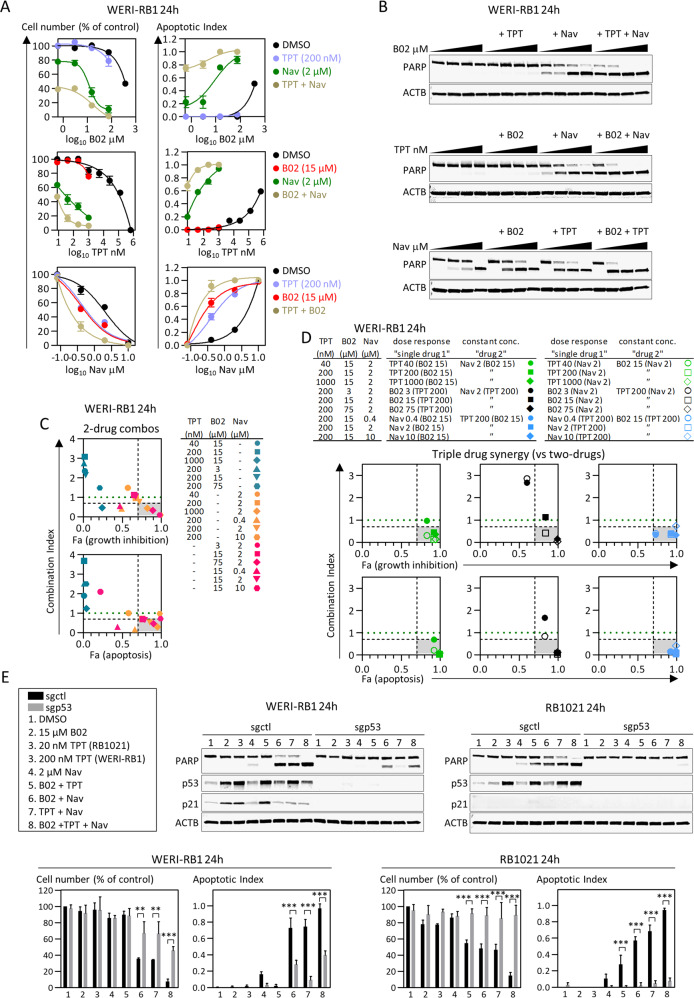
Synergistic drug combinations to kill B02/TPT-resistant RB. **a** WERI-RB1 were treated with increasing concentrations of B02 (top), TPT (middle), or Navitoclax (Nav, bottom), and with vehicle (black), or with EC50 doses of TPT (blue, two-drug combos), B02 (red, two-drug combos), or Nav (green, two-drug combos), or pairs of EC50 drugs (brown, three-drug combos), and cell number (**a**, left graphs) and apoptosis (**a**, right graphs) defined after 3 days (*n* = 2 ± range). **b** Representative PARP western used to quantify apoptosis in (**a**). The two-drug (**c**) or three-drug (**d**) combos from (**a**) are summarized in the tables, and CI vs. effect (Fa) graphed (gray areas and green lines as in Fig. 4a). **e** The indicated RB cell lines were transduced with control or p53-targeting sgRNA lentivirus, selected in puromycin, then 7 × 10^5^ cells were seeded and treated as indicated. At 24 h, cell number (trypan blue) and apoptosis (PARP) were quantified (*n* = 3, mean ± SD, **p < 0.01, ***p < 0.001 ordinary one-way ANOVA, Tukey’s multiple comparisons test). Representative PARP, p53, and p21 western blots are shown.

Next, we pursued a pharmaceutical strategy to bypass resistance to B02 + TPT. Our data reveal that, in all contexts, synergy engages proapoptotic BCL family proteins. Thus, we tested whether combining an inhibitor of antiapoptotic BCL proteins with B02 + TPT might overcome resistance. In addition, B02 or TPT alone activate p53 (Fig S16A, Fig. 5e, k), thus pairing either with a BCL inhibitor should also engage the BAX axis and drive synergy. Navitoclax (ABT-263) is an oral, bioavailable small molecule inhibitor of BCL2, BCL2L1, and less potently, BCL2L2 (BCL-w) [60]. We ran dose-response curves for Navitoclax, TPT, or B02 alone or in combination with fixed subEC50 doses of one (two-drug combos) or the other two drugs (three-drug combos). B02 + TPT combos had no effect, confirming resistance, but multiple TPT + Nav and B02 + Nav dual combos synergistically killed WERI-RB1 cells (Fig. 6a–c). Triple combos were even more effective in all three dose-response curves (Fig. 6a, b). To quantify triple drug synergy we considered a drug pair as single entity, thus the pair is kept constant and the third drug is varied [53, 61], and indeed many triple drug combos were synergistic relative to two drugs (Fig. 6d). Finally, we showed that either B02 + Nav or TPT + Nav dual therapies were also effective in RB1021 cells, that triple therapy further enhanced apoptosis in both RB lines, and that p53 was essential for this effect with all these drug combinations (Fig. 6e). Each therapy induced p21 in WERI-RB1 but not RB1021 cells, indicating that Navitoclax overrides this defense (Fig. 6e). Navitoclax is thus a new pharmacological agent for RB that synergizes with TPT and/or B02 to bypass p53-induced cell cycle arrest and resistance, and instead engage p53-driven apoptosis. The triple therapy may be generally applicable to most RB tumors irrespective of the dominance of the p53–BAX vs. p53–p21 axes.

## Discussion

Coupling dynamic network analysis with functional genomics, we uncovered new synergistic therapies for a pediatric cancer. Network analysis prioritized 61 candidates for an in vivo shRNA screen in orthotopic xenografts of two *RB1*^*null*^ lines, which were compared with 86 controls. Validation screens were run with 21 primary hits and 34 controls in three *RB1*^*null*^ lines and an *RB1*^*wt*^*;MYCN*^*amp*^ line, yielding 15 genes that were positive in both screens. These high value hits included known RB drivers (e.g., SKP2, MYCN, ID2), validating the screen, but also the RAD51/BRCA1 complex and various interacting partners. BRCA1 is a well-known tumor suppressor, but also has oncogenic roles with RAD51 through its DNA-repair function [62, 63]. We excluded other BRCA1 functions, but implicated BRCA1 and RAD51 in maintaining DNA integrity in RB cells. RB cells, but not normal fetal progenitors, underwent apoptosis upon RAD51 depletion. While cultured RPC are not a normal counterpart to RB cells, they provide an example of a proliferating *RB1*^*wt*^ cell type that is insensitive to combined RAD51- and topoisomerase-inhibitor therapies. To test the role of pRB loss in sensitizing to this combination it may be of interest to define B02 + TPT effects on *RB1*^*wt*^;*MYCN*^*amp*^ RB cells. The selectivity for RAD51 loss in *RB1*^*null*^ RB cells vs. RPC was reproduced with B02, a small molecule RAD51 inhibitor. Furthermore, B02 synergized with TPT or ETO, front line drugs in RB treatment, and these combinations also did not affect human RPC survival.

These positive results led us to test B02 + TPT in vivo. We observed synergistic efficacy in Y79 and RB1021, but not WERI-RB1 xenografts. These assays were performed over 7 days and with a vitreal model of disease, thus longer-term studies should be carried out prior to clinical tests, and additional models are required to examine effects on retinal disease. Mechanistically, B02 + TPT engaged the p53–BAX apoptotic axis in sensitive RB cells, but a protective p53–p21 G1-arrest pathway in drug-resistant cells. Deleting p21 switched the p53 response to engage BAX, driving apoptosis. That result suggested that BCL family inhibitors might bypass resistance, and indeed the BCL2/BCL-XL (BCL2L1)/BCL-W (BCL2L2) inhibitor Navitoclax synergized with either B02 or TPT, and the triple therapy was even more potent. This approach was effective with either B02 + TPT-sensitive RB1021 or B02 + TPT-resistant WERI-RB1 cells. Thus, Navitoclax warrants further testing as a new strategy to enhance TPT-killing of RB tumors, and RAD51 inhibitors could further heighten efficacy.

The synergy observed with TPT, B02, and/or Navitoclax in RB suggests these combinations may be effective in other p53^wt^ *RB1*-deficient cancers. Whether they could be exploited in *RB1/p53*-deficient cancers, such as small cell lung cancer (SCLC) or triple negative breast cancer (TNBC) is unclear. Recently, synergistic effects have been described in TNBC using CDC25 inhibitors plus WEE1 or PI3K inhibitors [64], as well as a synthetic lethal interaction between *RB1*-deficiency and Aurora Kinase A or B inhibitors in SCLC or TNBC [65, 66]. It will be interesting to compare the efficacy of these approaches with the TPT/B02/Navitoclax strategies outlined here in RB. A recent study found upregulation of BCL2 and WEE1 kinase in small cell neuroendocrine (SCN) cancers, and combining Navitoclax and the WEE1 inhibitor AZ-1775 was synergistic against SCN prostate cancer patient-derived xenografts [67]. Navitoclax was also partially successful in a phase II clinical trial for SCLC, with 23% of patients (9/39) showing stable disease and one patient with a partial durable response [68]. A drawback was the high rate of thrombocytopenia, which would not be a risk with localized IVT injection for RB. Follow-up work revealed an unusually high level of BCL2 in many SCLC tumors relative to most other solid cancers, which correlated with sensitivity to the BCL2-selective inhibitor, Venetoclax [69]. Thus, BCL inhibitors, especially in combination with synergistic drugs, may enhance therapeutic outcome in highly lethal RB-deficient SCN cancers.

Although dual combinations were potent, the most effective mix was the triple combination of TPT, B02, and Navitoclax. B02 has not been tested in the clinic, and as a single agent high µM concentrations are required, limiting its usefulness in vivo. Of note, CYT-0851 is another RAD51 inhibitor that was recently approved for a phase 1/2 clinical study in various cancers (NCT03997968) after showing promise in preclinical models [70], thus may be a potential valuable alternative. We observed potent in vitro synergy in triple therapy with 15 µM B02, and although we did not test this combination in vivo, B02 enhanced the response to TPT in preclinical trials (Fig. 4). Intravenous pharmacokinetics and toxicology cannot predict IVT or intra-arterial drug behavior, so preclinical studies are needed to assess clearance and toxicity in the eye with these modalities. Since TPT is currently used to treat RB, and Navitoclax has been approved for use in humans, the first priority will be to further test this combination in preclinical models and, if effective and safe, to bring this combination to the clinic.

## Materials and methods

### RNA and microarrays

Human fetal retinas were obtained from the Morgentaler Clinic in Toronto with approval from the Research Ethics Board (REB #13-0132-E) of Mount Sinai Hospital in Toronto, Canada, and consent from patients. Gestational age was estimated by a combination of clinic intakes, ultrasound, crown-rump, and fetal foot length measurements where possible [71, 72]. Eye samples collected were held on ice for up to 6 h in the retina culture medium (IMDM with 10% FBS and 1× Antibiotic–Antimycotic (Life Technologies, Toronto, ON, Canada)). RB samples were obtained at the Vision Research Foundation, Sankara Nethralaya Chennai, India, with the approval from the institutional research and ethics board, and consent from patients. RNA quality was determined using Agilent 2100 Bioanalyzer and only medium-high quality samples were processed. RNA was reverse transcribed and hybridized to Illumina Human V6 beadchips (v2, San Diego, CA, USA). Murine RNA samples were collected using RNeasy Mini kit (Qiagen, Venlo, The Netherlands) from retina of mixed C57BL/6 × 129SvJ backgrounds. RNA quality was determined using Agilent 2100 Bioanalyzer (TCAG, Toronto, ON, Canada). Only samples with an RNA Integrity score of ≥7 were used. Reverse transcription and hybridization were performed by TCAG, Toronto. Samples were hybridized to Illumina Mouse WG6 beadchips (v1.1, San Diego, CA, USA). Probe intensity scores were processed using RMA background correction and log2 transformation.

### DyNeMo

We focussed on human/mouse orthologues present in the OPHID network. From 24,582 human and 13,544 mouse geneids we identified 10,633 homologous geneids. We mapped the geneids to the OPHID ppi skeleton (formed by the edges with the highest betweenness centralities of the origin network, which is believed to preserve the modular structure of biological network while greatly simplifying the complexity of the highly entangled origin network), of which 4480 remained after removing singletons. We first ran DyNeMo on 21 human RB transcriptomes vs. 12 normal human fetal retina (4 × week 12, 4 × week 15, 4 × week 18) transcriptomes. Three DyNeMo runs were performed, with each run using a randomly selected two-thirds of the samples (14 tumors, 8 normals). We selected hubs that were significant in all three runs by nonparametric *p* values, generated by comparing the mean absolute difference of the Pearson correlation coefficient in the tumor/normal samples versus 1000 randomly generated samples. To obtain evolutionarily conserved hubs, we repeated DyNeMo with transcriptome data from 14 normal murine retinal samples (3 E15.5, 3 P0, 4 P8, 4 adult/1mo) and 23 murine RB samples, harvested when the affected adult eye was full of tumor, from 5 mouse genetic models (5× *αCre;Rb*^*f/f*^;*p107*^*−/−*^, 4× *Chx10Cre;Rb*^*f/f*^;*p107*^*−/−*^, 4× *αCre;Rb*^*f/f*^;*p27*^*−/−*^, 3× *αCre;Rb*^*f/f*^;*p27*^*CK-/CK-*^, 3× *αCre;Rb*^*f/f*^;*p130*^*−/−*^, 4× TAg-RB). All animal experiments were conducted with ethical approval from the animal care committees of each research institute (University Health Network for all models, except *αCre;Rb*^*f/f*^;*p130*^*−/−*^, in which case mRNA was a gift of David Macpherson at the Fred Hutchinson Cancer Research Centre, Seattle, USA; TAg-RB samples were a gift from Brenda Gallie). The models have been described previously [73–77].

### RNAi screens and validation

A custom pool of bar-coded shRNAs in the pLKO.1 lentiviral backbone targeting DyNeMo candidate hubs (detailed in Table S1) was first cherry picked from The RNAi Consortium (TRC) library, then pooled lentiviruses were produced according to standard protocols (https://portals.broadinstitute.org/). In the primary screen, WERI-RB1 or Y79 cells were transduced at MOI = 0.2 and selected in puromycin. T0 genomic DNA (gDNA) was isolated from some of the cells, and the remainder was orthotopically transplanted into NOD-SCID eyes (250,000 per eye). Six tumors were grown per cell line. After large tumors formed (4–6 weeks) deep sequencing was performed and revealed 0.1–27,000 normalized reads per virus, but ~95% shRNAs yielded ≥ 10 normalized reads (Fig. S2) [78]. Outlier exclusion was performed prior enrichment analysis. Briefly, normalized reads were log transformed for data linearization then robust *Z*-scores for each shRNA in every sample were obtained, allowing outlier identification (|robust *Z*| > 5) and exclusion of ≤2/6 tumors per shRNA for each cell line, leaving ≥ 4 tumor replicates per shRNA for enrichment/depletion analysis. Next, averaged tumor/T0 counts for each shRNA minus outliers were obtained and log transformed. Fold-change was then converted into *Z*-scores after confirming that the distribution of log tumor/T0 dataset followed a Gaussian pattern (Fig. S2D, Table S1). For the second screen with four RB cell lines, hit shRNAs from screen 1 as well as an equal number of nonscoring shRNAs were selected (detailed in Table S1). The same procedure was followed to generate pooled viruses, transduce cells, and collect T0 and tumor gDNAs. The generated data were not Gaussian, reflecting the reduced n and high hit fraction in the second screen, thus *Z*-scores could not be calculated, so a stringent cutoff of fivefold reduced tumor growth (log ratio −0.69) and ≥2 scoring shRNAs was used to establish robust hits (shaded area in Fig. 2d). Hits were further classified as high, medium, or low quality if they scored in ≥3, 2, or 1 of the 4 cell lines, respectively (Fig. 1f, Table S1).

For validation, Y79 cells were transduced with shRNAs viruses scoring in both screens. Knockdown efficiency was assessed by western blot, and cell number by trypan blue. The following shRNAs from TRC shRNA library were used: luciferase (negative control) TRCN0000072256; BRCA1 #1 TRCN0000244985, BRCA1 #2 TRCN0000244986; RAD51 #1 TRCN0000018876, RAD51 #2 TRCN0000329688; SKP2 #1 TRCN0000007534, SKP2 #2 TRCN0000315078; PABPC1 #1 TRCN0000074639, PABPC1 #2 TRCN0000074641; MSI #1 TRCN0000062811, MSI #2 TRCN0000062812.

### Cell culture

Dissociated RPC were cultured in Iscove’s modified Dulbecco’s medium supplemented with 10% fetal bovine serum (FBS), 1× 2-mercaptoethanol (Gibco, ThermoFisher Scientific, Burlington, ON, Canada), 10 μg/ml plasmocin, and 1× anti–anti (Gibco). The low passage RB cells lines RB3823, RB3535S, RB4036, RB4063, RB3935 were grown in T25 flasks in Iscove’s medium supplemented with 10% FBS, antibiotics, 0.0004% (v/v) 2-mercaptoethanol, and 10 μg/L insulin as previously described [79]. We cultured RB1021 cells (gift from Brenda Gallie) in the same conditions as the low passage RB lines, WERI-RB1 and Y79 cells were cultured in RPMI1640, A549 cells in DMEM, H661 in RPMI1640, PFSK-1 (gift from Dr. Annie Huang) in DMEM with 1× MEM NEAA (Gibco), and all were supplemented with 10% FBS and antibiotics. Cell lines were mycoplasma free. We performed STR analysis on four of the RB lines, two of which have STR profiles (Y79, WERI-RB1) and two of which do not (RB3823, RB1021), so the latter will be useful to others in the future; the information is provided in Table S2. The STR profiles for Y79 and WERI-RB1 were 97 and 84% matches and designated as “derived from a common ancestor” by the test lab (The Centre for Applied Genomics, Hospital for Sick Children, Toronto, Canada), consistent with modest drift from the original source. For the other early passage cell lines mentioned in the westerns for Fig. 1g, those lysates were a gift from Dr. Brenda Gallie (Hospital for Sick Children, Toronto). Dr. Gallie was routinely generating and genotyping tumor lines at that time and is heavily involved in genotyping patients/tumors, thus we relied on their considerable expertise to assure authenticity.

### Western blotting

Cells were lysed for 1 h on ice in RIPA buffer (Santa Cruz, Mississauga, ON, Canada) supplemented with protease inhibitor cocktail, sodium orthovanadate and PMSF. Lysates were run on 4–20% SDS-PAGE gradient gels, transferred to nitrocellulose, and analyzed by Li-Cor system (LI-COR Biosciences, Lincoln, NE, USA) with antibodies listed in Table S3.

### Cell growth assays

2D culture; 96-well format: RB cells were seeded at 10,000 cells/100 µl. MDA-MB-231 cells were seeded at 3000 cells/100 µl. Drugs were prepared by serial dilutions at 6× concentrations and 20 μl were added to the cells. Cell viability was assessed at different timepoints with CellTiter-Glo Reagent (Promega, Madison, WI, USA), and luminescence quantified with a plate reader (EnVision, PerkinElmer, Woodbridge, ON, Canada). Six-well format: RB cells and RPC were seeded at 700,000 and 300,000 cells/2 ml, respectively. The next day (day 0 of the assay), drugs, siRNAs, or doxycyclin were prepared at 5× and 0.5 ml added to cells. Cell counts were performed with trypan blue.

3D culture; 96-well format: each well was first plated with 50 μl of medium containing 0.6% agar, then Y79 cells were seeded at 600 cells/60 μl medium containing 0.35% agar. Drugs were prepared at 2.8× and 60 μl added on top of the cell layer. After 6 days, colonies were assessed with Alamarblue Cell Viability Reagent (ThermoFisher, Burlington, ON, Canada), as described [80], and absorbance quantified with a plate reader as above.

### siRNA-mediated gene silencing

Cells were seeded in six-well plates in medium without antibiotics. RB and PFSK-1 cells were seeded at 700,000 cells/2 ml, and A549, H661 cells at 200,000 cells/2 ml. siRNA mixes with DharmaFECT 1 Transfection Reagent (Horizon Discovery) were prepared as per the manufacturer’s instructions at a final concentration of 50 nM unless specified otherwise. Mixes were added at day 0 (and day 3 if assay > 3d, and cells were passed if >80% confluent). siRNAs were from Qiagen: negative control SI03650318; BRCA1 SI02654575, SI02664368; RAD51 SI00045010, SI02629837; and Dharmacon siGenome: p53 D-003329-07.

### Re-expressing RAD51 in siRAD51-treated cells

RAD51 was re-expressed using a tetracycline-inducible system (Takara Bio, Mountain View, CA, USA). Briefly, RAD51 cDNA fused with Flag in C-terminal was cloned into the pLVX-TRE3G-mCherry vector and empty or RAD51 pLVX-TRE3G-mCherry lentiviruses as well as pLVX-Tet3G viruses were produced in 293T cells. Y79 cells were then sequentially transduced with the Tet3G virus, selected with 1 mg/ml G418 for 7 days, then transduced with the second empty or RAD51 TRE3G-mCherry virus, and selected with 3 μg/ml puromycin for 7 days. RAD51 expression was induced with 2 μg/ml doxycyclin and cells were treated with a control or siRNA targeting the 3′ UTR region of RAD51 24 h later.

### Cell fractionation

Protein lysates from 500,000 cell equivalents were prepared with the subcellular protein fractionation kit for cultured cells (ThermoFisher, Burlington, ON, Canada). Total protein lysates were extracted in RIPA buffer (see “Western blot” section).

### Fluorescence microscopy

#### Cultured cells

In six-well plates, coverslips were precoated with 50 µg/ml of poly-d-lysine (Sigma, Markham, ON, Canada) and 700,000 RB cells and 300,000 dissociated RPC were seeded in 2 ml/well overnight. Where applicable, cells were labeled with MitoTracker Deep Red FM (Cell Signaling, Whitby, ON, Canada) or EdU as per manufacturer’s instructions. Cells were fixed with 4% paraformaldehyde for 10–15 min, permeabilized and blocked for 1 h, then subjected to click chemistry and probed with primary antibodies overnight at 4 °C as in Table S3. After 2–3 washes, cells were probed with Alexa Fluor 488- or 568-conjugated secondary antibodies (ThermoFisher, Burlington, ON, Canada) and 4,6-diamidino-2-phenylindole or propidium iodide (PI) for 90 min. Coverslips were mounted using VectaShield (Vector Laboratories, Burlington, ON, Canada). High resolution confocal images were acquired with the Wave FX Spinning Disc Confocal microscope (Quorum Technologies, Puslinch, ON, Canada) and Volocity software.

#### Cryosections

Mice were i.p. injected with 10 mg/kg EdU for 1 h before sacrifice. Frozen eye sections were blocked and permeabilized in PBS with 5% donkey serum and 0.1% Tween20 for 1h30 at RT. Slides were incubated with the click reaction mix, and antibody staining performed as described [74, 81].

### Cell cycle, EdU/DNA staining

Cells in six-well plates were labeled using the Click-iT EdU Alexa Fluor 647 Flow Cytometry Assay Kit then counterstained for DNA with FxCycle Violet dye (ThermoFisher, Burlington, ON, Canada). At least 10,000 single cells per sample were acquired using the Gallios flow cytometer (Beckman Coulter, Mississauga, ON, Canada) and analysed with Kaluza software.

### High throughput detection of DSBs in the cell cycle

Y79 cells were processed as described in the *Edu/DNA content staining* section and after the click chemistry step the cells were probed with γH2A.X antibody in blocking/permeabilization buffer overnight at 4°. The cells were then washed 2× in PBS with 0.1% saponin for 10 min, probed with Alexa Fluor 488-conjugated secondary antibody at 1/500 dilution (ThermoFisher Scientific, Burlington, ON, Canada) for 90 min at RT rotating, washed 2×, and stained with 1.5 µg/ml FxCycle™ Violet dye for 1 h. At least 10,000 single cells per sample were acquired with the Amnis ImageStreamX Mark II Imaging Flow Cytometer to detect nuclear γH2A.X foci per cell in the different phases of the cell cycle. Samples were analyzed with the IDEAS v6.2 software.

### Annexin V and FxCycle Violet staining

Live cells were washed with PBS and resuspended in 150 µl staining buffer with alexa Fluor^®^ 488 annexin V (ThermoFisher, Burlington, ON, Canada) and 1.5 µg/ml FxCycle Violet dye for 25 min at RT. At least 10,000 single cells per sample were acquired using flow cytometry as above.

### Mitochondrial oxidative stress

Cells were labeled with Mitosox red mitochondrial superoxide indicator (ThermoFisher Scientific, Burlington, ON, Canada) as per manufacturer’s instructions. Fluorescence intensity was quantified and analyzed by flow cytometry as above.

### Bright field images

Hematoxylin and eosin (H&E)-stained sections were captured with the Olympus BX61 microscope, and cultured cells were captured with the Zeiss Axio Vert.A1 microscope.

### Compounds

TPT hydrochloride (HPLC: 99% purity, diluted in DMSO for in vitro assays and sterile PBS for in vivo assays), ETO (HPLC: 99% purity, diluted in DMSO), Navitoclax (HPLC: 98% purity, diluted in DMSO), PF-477736 (HPLC: 99% purity, diluted in DMSO), MK-8776 (HPLC: 96.4% purity, diluted in DMSO), KU-55933 (HPLC: 99% purity, diluted in DMSO), 3-methyl adenine (HPLC: 99% purity, diluted in sterile water), and wortmannin (HPLC: 99% purity, diluted in DMSO) were purchased from Selleckchem (Cedarlane, Burlington, ON, Canada). The RAD51 inhibitor B02 (HPLC: 98% purity, diluted in DMSO for in vitro assays and sterile PBS for in vivo assays) was purchased from Calbiochem (Millipore, Etobicoke, ON, Canada), the CHK2 inhibitor II (HPLC: 98% purity, diluted in DMSO) from Sigma (Markham, ON, Canada), and Trolox (HPLC: 97% purity, diluted in DMSO) from Santa Cruz (Mississauga, ON, Canada).

### CRISPR-mediated gene knockout

sgRNA sequences were cloned into the LentiCRISPR v2 vector (Addgene Plasmid #52961), or for double knockout cells, sgControl or p21 sgRNA #2 were cloned into a modified version where the puromycin resistance gene was replaced with hygromycin. Guide sequences were as follow: sgctl: CGCTTCCGCGGCCCGTTCAA; p21 sgRNA #1: TCAGAACCCATGCGGCAGCA, p21 sgRNA #2: GTCACCGAGACACCACTGGA; p53 sgRNA: CCATTGTTCAATATCGTCCG. Briefly, we transduced WERI-RB1 cells with the indicated sgRNA viruses. Four days after transduction, cells were selected in 1 µg/ml puromycin for 5 days to generate stable knockouts. To generate double knockout cells, sgControl or p53 knockout cells were transduced with an sgControl (hygromycin) or sgp21 (hygromycin) virus. Four days after transduction, cells were selected in 50 µg/ml hygromycin B for an additional 6 days.

### Synergy determination

CI and DRI were computed with CompuSyn software based on the effects on either growth inhibition or apoptosis. The effect-oriented Fa–CI plot was used to represent the data because a normalized isobologram would be over-crowded: Fa (growth inhibition), or Fa on growth inhibition, represents the inhibitory effect of a combo on cell number, and is expressed as 1 − (*X*_combo_/*X*_ctl_), where *X* is cell number, and therefore a value of 0 means no effect compared to control, whereas 1 equals complete growth inhibition; Fa (apoptosis) represents the apoptotic index, a value of 0 means no apoptosis, whereas 1 equals complete apoptosis [53]. We increased stringency to highlight the most potent combinations based on three criteria: (1) Fa > 0.7 in the combo, reflecting major enhancement; (2) CI < 0.7, reflecting synergy; and (3) Criteria #1, 2, met whether we varied BO2, TPT, or ETO. Criteria (1) and (2) delineate a shaded area on the Fa/CI plots representing the therapeutically relevant synergy. Synergy of three-drug combinations was performed as previously described [61].

### Comet assay

Cells were processed for the alkaline comet assay (detection of DSBs) according to the manufacturer’s instruction (Trevigen, Cedarlane, Burlington, ON, Canada). DNA comets were stained with 1.5 μg/ml PI for 1 h at RT in the dark. At least 300–400 nucleoids (normal or comet) per assay were quantified with the Wave FX Spinning Disc Confocal microscope (Quorum Technologies, Puslinch, ON, Canada)

### Efficacy in orthotopic xenograft

Animal protocols were in accordance with local and national guidelines. IVT injections were performed under general anesthesia using isoflurane. Human RB cell lines stably expressing luciferase were prepared at 25,000 cells/μl in sterile PBS with 10% matrigel (BD Bioscience, San Jose, CA, USA) and 5% trypan blue, then 2 μl of the mixture were injected into the right vitreous of 3–4 weeks old male NOD-Scid mice. Uninjected left eye served as a negative control. After seven days, B02, Topotocan, a mix of B02 + TPT, or vehicle (PBS) was injected randomly in tumor-bearing eyes at the indicated doses (three animals/dose unless specified otherwise). Luc^+^ tumor cells were tracked live by i.p. injection of d-luciferin at 150 mg/kg for 10 min and radiance total flux (photons/second) tracked with the Xenogen IVIS Imaging System 100 (PerkinElmer, Woodbridge, ON, Canada). At each timepoint, biological replicates were normalized to day 0, averaged, and plotted. Data acquisition was blinded.

### Eye toxicity

B02 was injected in the vitreous of anesthetized 3–4 weeks old Nod-Scid mice at 0.3, 3, 10, 30 µg, and 3 days later the mice were sacrificed, the eyes enucleated and incubated in Davidson’s fixative overnight at 4 °C on a shaker. Ethanol dehydration and paraffinization of the tissue were done in a tissue processor (Excelsior ES, Thermo Scientific, Burlington, ON, Canada). Sections (5 µm) were prepared using an ultramicrotome (Leica Microsystems, Richmond Hill, ON, Canada). Sections were deparaffinised and rehydrated before staining with H&E.

### Statistical analysis

GraphPad Prism 8 statistical software (San Diego, CA, USA) was used to calculate EC50s, perform unpaired *t*-test (two-tailed *p* values), two-way ANOVA with Sidak’s multiple comparisons test, and ordinary one-way ANOVA with Tukey’s multiple comparisons test.

## Supplementary information

Supplementary Figures 1 to 19

Table S1

Table S2

Table S3

## Data Availability

DyNeMo algorithm was generated previously [21].
