# Supplementary material for: Functional genomics identifies new synergistic therapies for retinoblastoma
Source: Oncogene. 2020 Jun 22;39(31):5338–57. doi: 10.1038/s41388-020-1372-7 (PMC7391301; doi:10.1038/s41388-020-1372-7)
Supplement: Supplementary file 1 — Supplementary Figures 1 to 19 [file 41388_2020_1372_MOESM1_ESM.pdf]

A

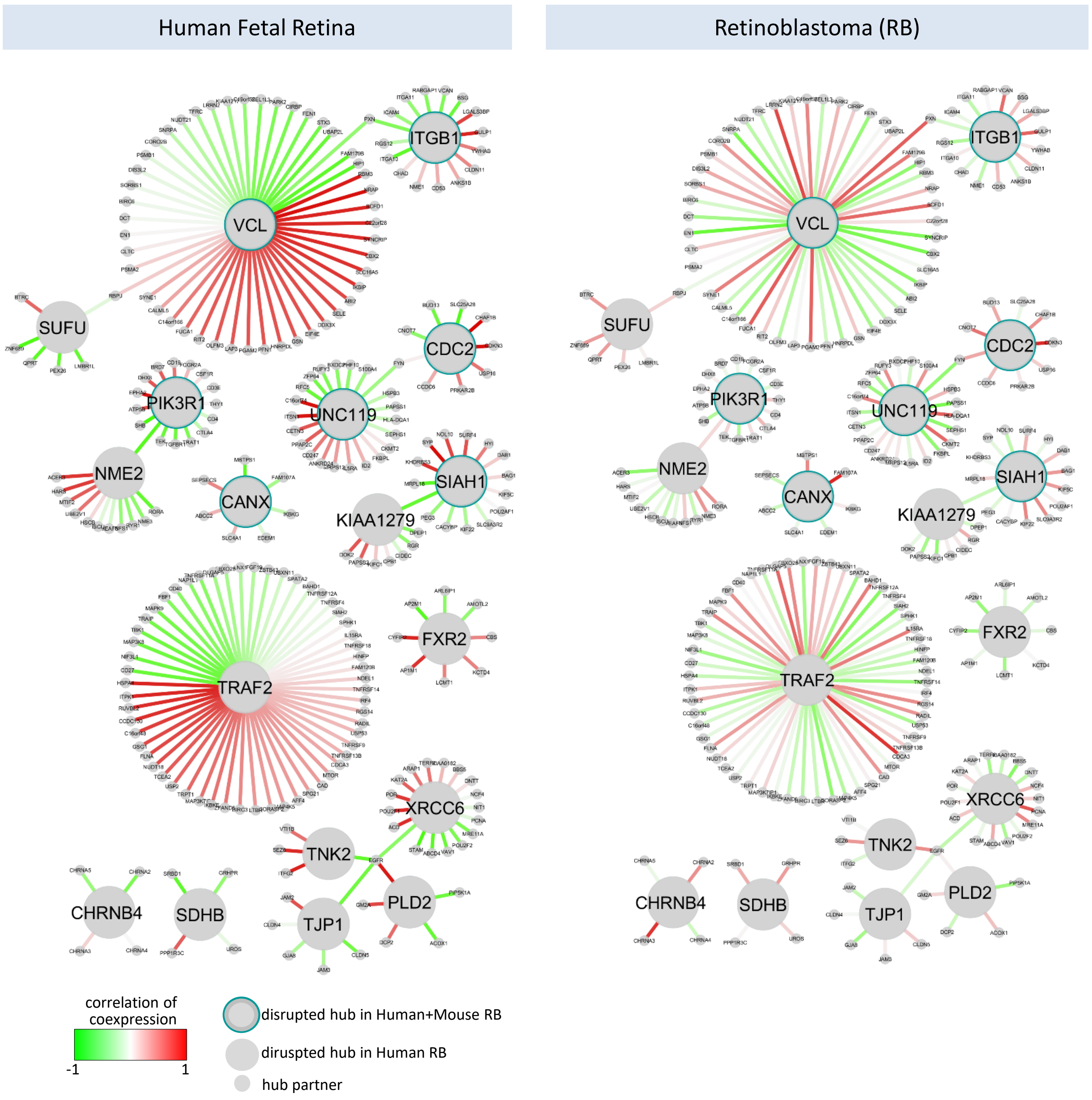

B

Disrupted Hubs in Human and Mouse RB		Disrupted Hubs in Human RB	
CANX	SIAH1	BRCA1	SDHB
CDC2 (CDK1)	SRC	CHRNB4	SUFU
ITGB1	SRF	FXR2	TJP1
MAD2L1	UNC119	KIAA1279	TNK2
PIK3R1	VCL	NME2	TRAF2
PRKCZ		PABPC1	TRIP13
		PLD2	XRCC6
		RAD51	ZBTB16

Figure S1. Identification of novel retinoblastoma therapeutic candidates using DyNeMo.
A Network of the interacting partners of hubs that are disrupted in RB tumors *vs.* Human Fetal Retina. Edges indicate correlation of expression between protein pairs. Full list of hubs and their corresponding partners and PCC values are available in **Table S1 "DyNeMo result"**. **B** Table summarizing the disrupted hubs identified by DyNeMo.

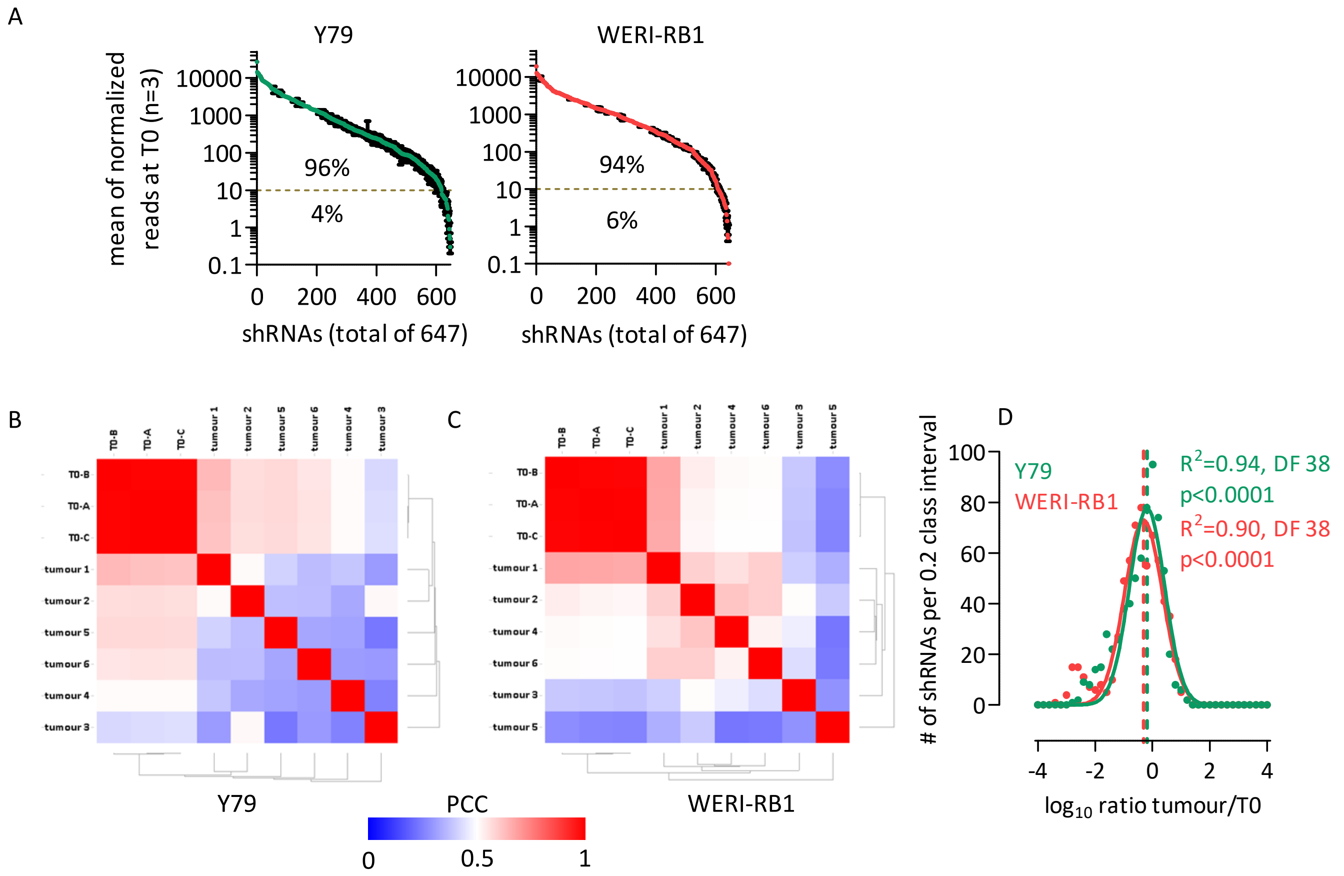

Figure S2. QC of the *in vivo* primary dropout screen.
A shRNA recovery in Y79 and WERI-RB1 in the primary screen. Quantified sequencing reads for each shRNA at T0 were ranked and plotted as mean +/- SD of 3 biological replicates. **B-C** Correlation heatmaps between T0 and tumor shRNA reads. The Pearson correlation coefficient (PCC) is shown for each pair comparison. **D** Distribution of the shRNAs $\log_{10}$ ratio tumor/T0 in the indicated RB lines. Gaussian fitting curves were computed using Graphpad Prism. R^2 indicates goodness of the Gaussian fit, DF the Degree of Freedom, and *p* values show significance.

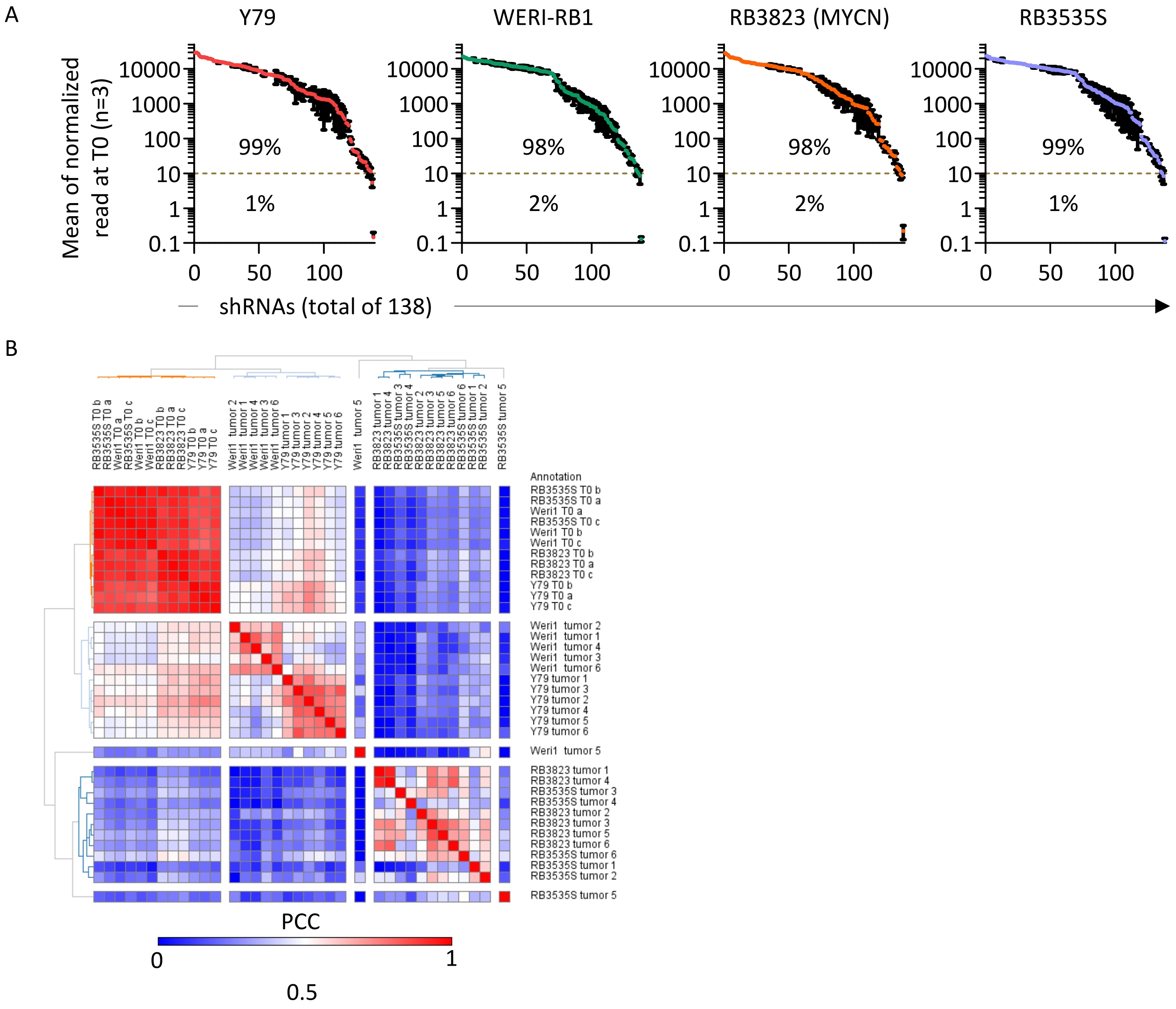

Figure S3. QC of the *in vivo* secondary dropout screen.

A shRNA recovery in the indicated RB lines in the secondary screen. Quantified sequencing reads for each shRNA at T0 were ranked and plotted as mean +/- SD of 3 biological replicates. **B** Correlation heatmaps between T0 and tumor shRNA reads in the secondary screen. The Pearson correlation coefficient (PCC) is shown for each pair comparison.

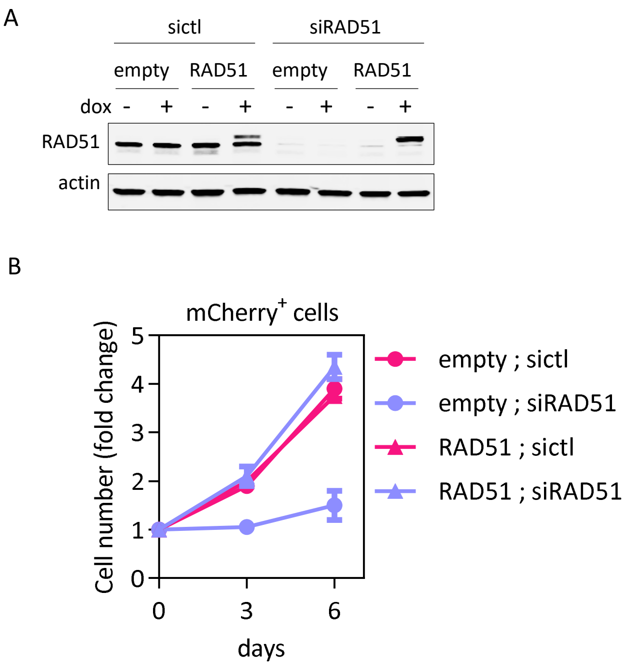

Figure S4. RAD51 expression rescues the growth defect in RAD51-depleted cells.
A Y79 cells were transduced with a Dox-inducible Empty/mCherry or RAD51/mCherry lentivirus, and selected in 7 µg/ml puromycin for 7 days. On day zero, cells were treated with 2 µg/ml doxycycline, then exposed to control or RAD51 siRNA 24h later, and RAD51 westerns run on day 6. **B** At time 0 and each indicated subsequent time point total cells were counted with trypan blue, then adjusted for the fraction that were mCherry^{+} using flow cytometry. All values were normalized to the starting amount on day 0 to determine the fold increase in mCherry^{+} cells in each condition (n = 2, mean +/- range).

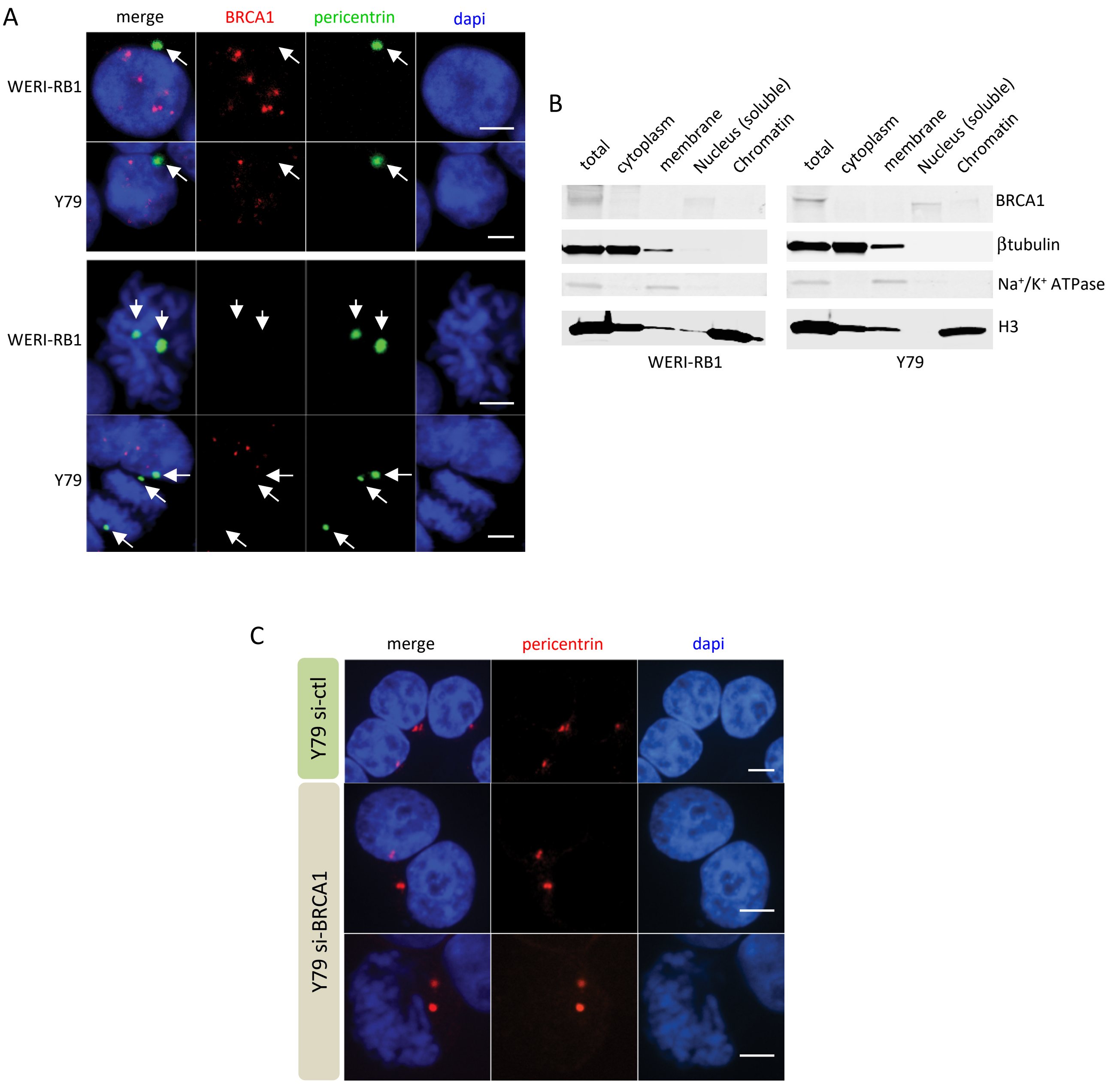

Figure S5. No effect of BRCA1 depletion on centrosome duplication.

A The indicated RB cell lines were immunostained for BRCA1 (red) and the centrosome protein pericentrin (green), and analyzed by confocal microscopy. Interphase (top panel) and mitotic (bottom panel) cells show nuclear BRCA1 foci in interphase only. Arrows indicate that BRCA1 does not co-localize with centrosomes. Magnification is 120x. **B** Subcellular fractionation followed by westerns in the indicated RB cell lines. Purity of the fractions was assessed by βtubulin for cytoplasm, Na^+/K^+ ATPase for membrane, and H3 for chromatin. **C** Y79 cells treated with siCtl or siBRCA1 were immunostained at day 5 for pericentrin and analyzed by confocal microscopy. Of > 10,000 cells assessed in two assays, none exhibited more than two centrosomes. Scale bars are 5 µm.

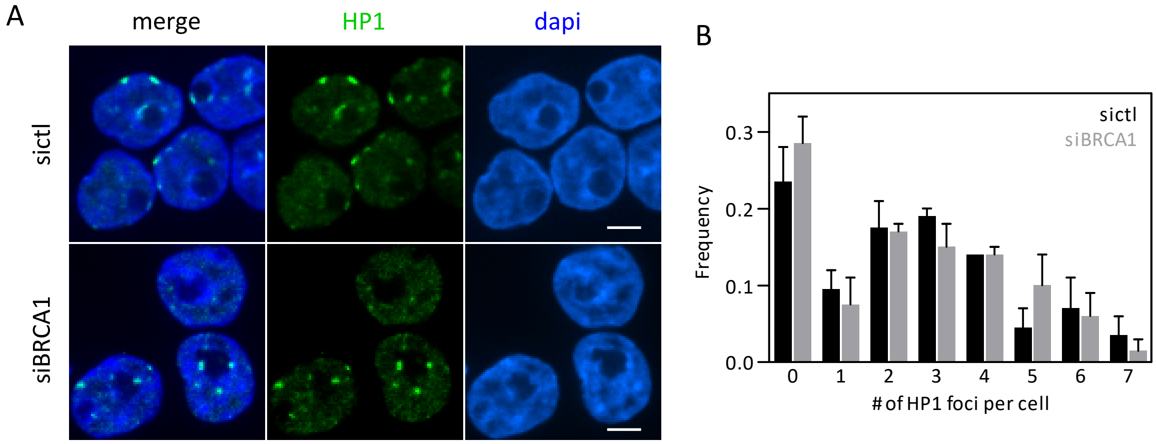

Figure S6. BRCA1 does not alter the heterochromatin marker HP1.
A Y79 cells treated with siCtl or siBRCA1 were immunostained at day 5 for HP1 (heterochromatin marker) and analyzed by confocal microscopy. Magnification is 120x. **B** Bar graph showing the distribution of HP1 foci per cell from (A) quantified from ≈ 150 cells (n = 2, mean +/- range). Scale bars are 5 µm.

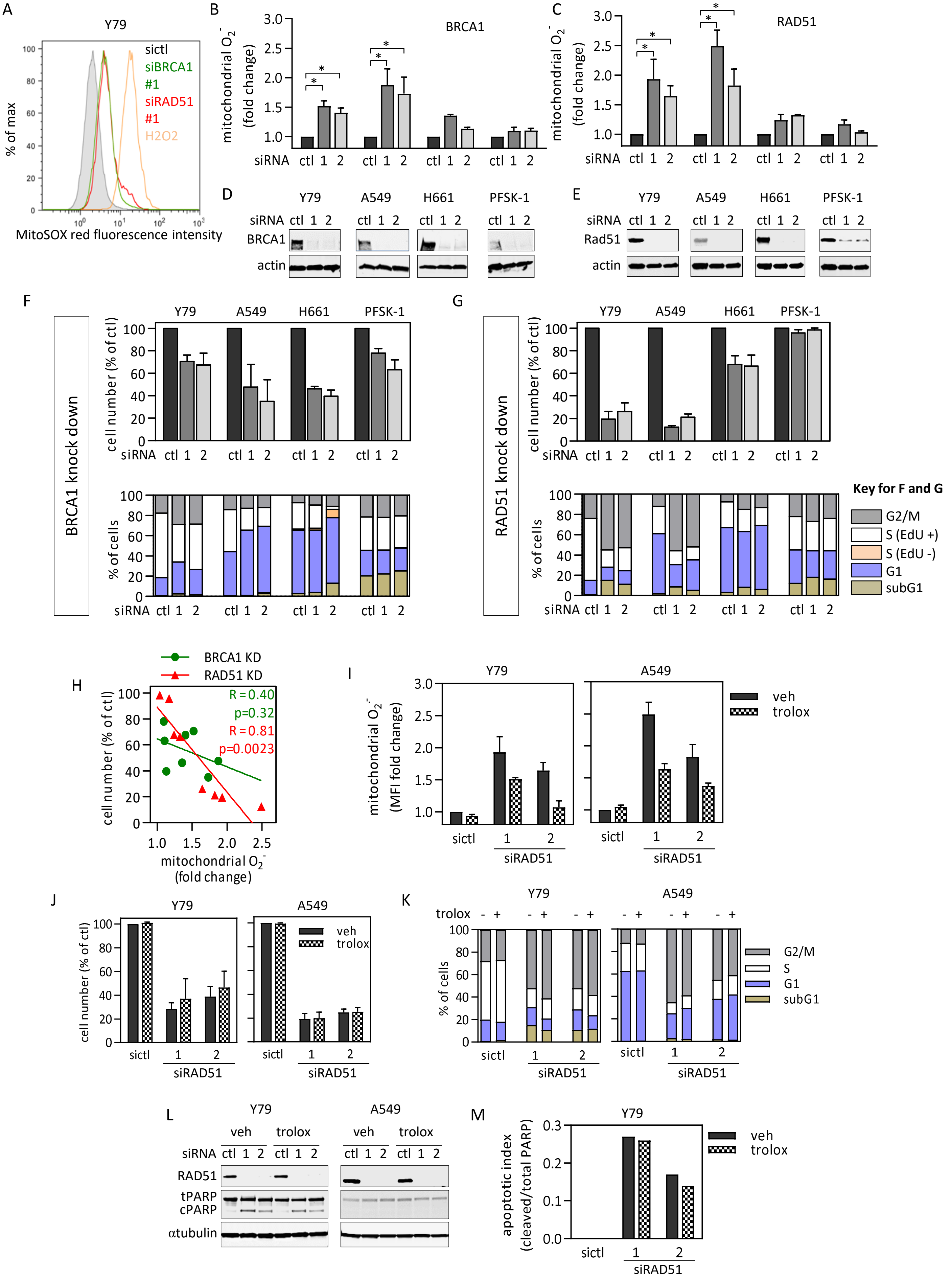

A
Y79
sictl
siBRCA1
#1
siRAD51
#1
H2O2
% of max
MitoSOX red fluorescence intensity
B
BRCA1
mitochondrial O2- (fold change)
siRNA ctl 1 2 ctl 1 2 ctl 1 2 ctl 1 2
C
RAD51
mitochondrial O2- (fold change)
siRNA ctl 1 2 ctl 1 2 ctl 1 2 ctl 1 2
D
Y79
A549
H661
PFSK-1
siRNA ctl 1 2
BRCA1
actin
E
Y79
A549
H661
PFSK-1
siRNA ctl 1 2
Rad51
actin
F
BRCA1 knock down
Y79
A549
H661
PFSK-1
cell number (% of ctl)
siRNA ctl 1 2 ctl 1 2 ctl 1 2 ctl 1 2
% of cells
G
RAD51 knock down
Y79
A549
H661
PFSK-1
cell number (% of ctl)
siRNA ctl 1 2 ctl 1 2 ctl 1 2 ctl 1 2
% of cells
Key for F and G
G2/M
S (EdU +)
S (EdU -)
G1
subG1
H
BRCA1 KD
RAD51 KD
R = 0.40
p=0.32
R = 0.81
p=0.0023
cell number (% of ctl)
mitochondrial O2- (fold change)
I
Y79
A549
veh
trolox
mitochondrial O2- (MFI fold change)
sictl
1
2
siRAD51
J
Y79
A549
veh
trolox
cell number (% of ctl)
sictl
1
2
siRAD51
K
Y79
A549
trolox - + - + - +
% of cells
G2/M
S
G1
subG1
sictl
1
2
siRAD51
L
Y79
A549
veh
trolox
siRNA ctl 1 2 ctl 1 2
RAD51
tPARP
cPARP
αtubulin
M
Y79
veh
trolox
apoptotic index (cleaved/total PARP)
sictl
1
2
siRAD51

Figure S7. BRCA1 or RAD51 depletion cause cell cycle and survival defects independent of mitochondrial ROS.

A-C The fluorescence intensity of Mitosox red, a mitochondrial superoxide indicator, was assessed by flow cytometry in the indicated cells treated with a control or 2 different BRCA1 and RAD51 siRNAs for 5 d. A representative plot of the flow cytometry data in Y79 is shown with 5 mM H_2O_2 for 1h as a positive control (A), and the Mean fluorescence intensity (MFI) of mitosox red, normalized to sictl, is graphed for 4 cell lines (n = 3, average fold change +/- SD, * $p < 0.05$ Student t-test). **D-E** Westerns confirming depletion of BRCA1 (D) and RAD51 (E). **F-G** Graphs of cell growth (top) and cell cycle (bottom) assessed at day 5 after BRCA1 (F) and RAD51 (G) depletion. **H** Graph of cell number (from (F-G)) *vs*. mitochondrial ROS (from B-C) for BRCA1 (green) or RAD51 (red) depletion. Linear regression fits were computed. Goodness of fit (R square) and significance (p value) are shown. **I-M** The indicated cell lines sensitive to RAD51 KD were treated with 50 nM siRAD51 alone (ctl) or together with the ROS scavenger Trolox (200 μM) for 6 days. The effect on mitochondrial ROS was assessed by flow cytometry and plotted as fold change to control in (I). Cell growth, assessed by trypan blue, was plotted as percentage of control (J). Cell cycle phases, assessed by flow cytometry after EdU/fxcycle staining, were plotted as percentage of total cells (K). Westerns were used to quantify RAD51 depletion and PARP cleavage (L**),** and the latter used to quantify apoptosis **(**M). Data is average of 2 assays +/- range.

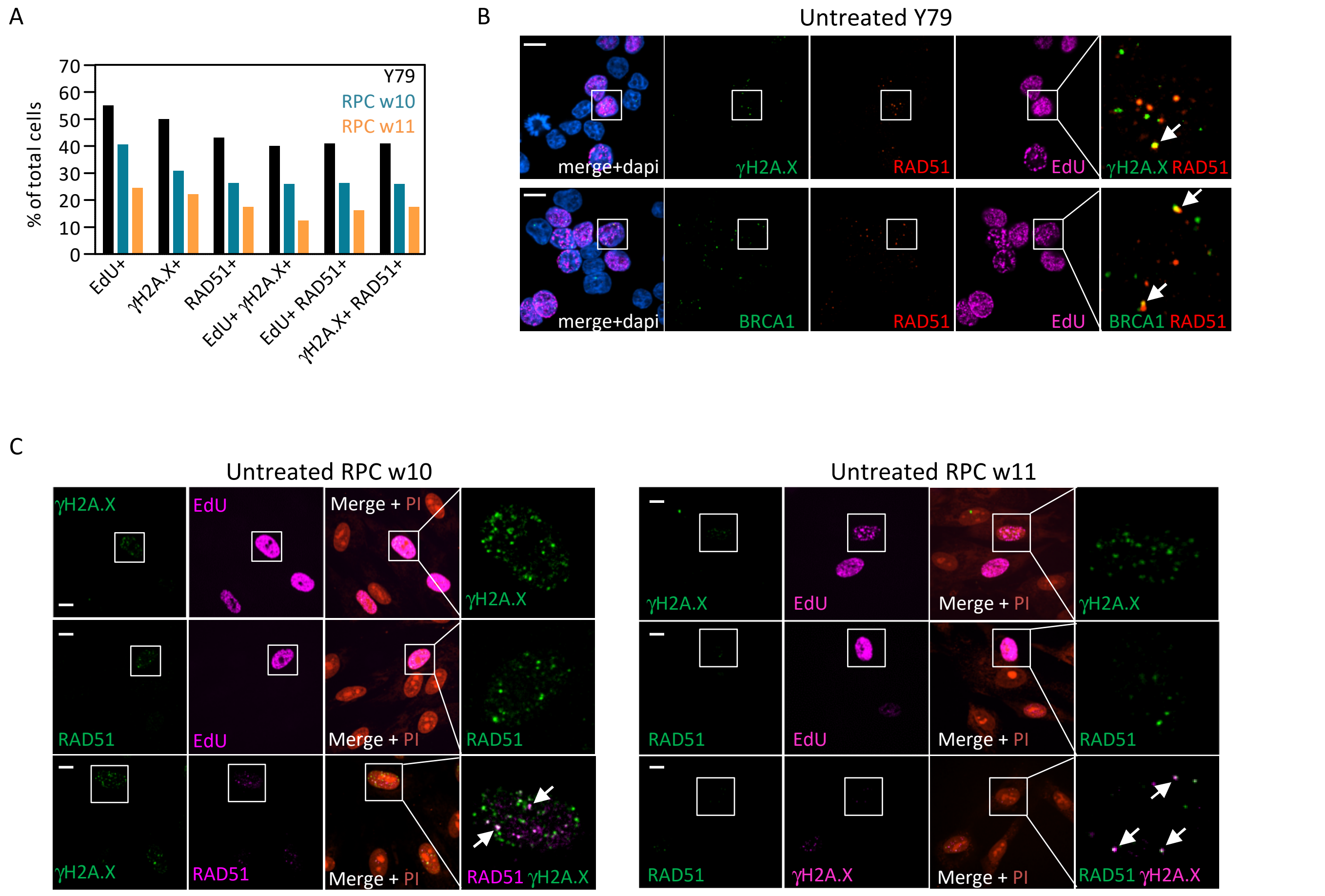

Figure S8. EdU-labeling and immunostaining of BRCA1, RAD51, and γH2A.X in untreated Y79 cells and RPC.
A-C Y79 (B) or two RPC (C) cell preparations were pulsed with EdU for 30 min to label S phase cells prior to fixation, click chemistry, and immunostaining for BRCA1, RAD51, and γH2A.X. Quantification (A) and examples of staining in Y79 cells (B) or RPC (C) are shown. Scale bars are 10 µm.

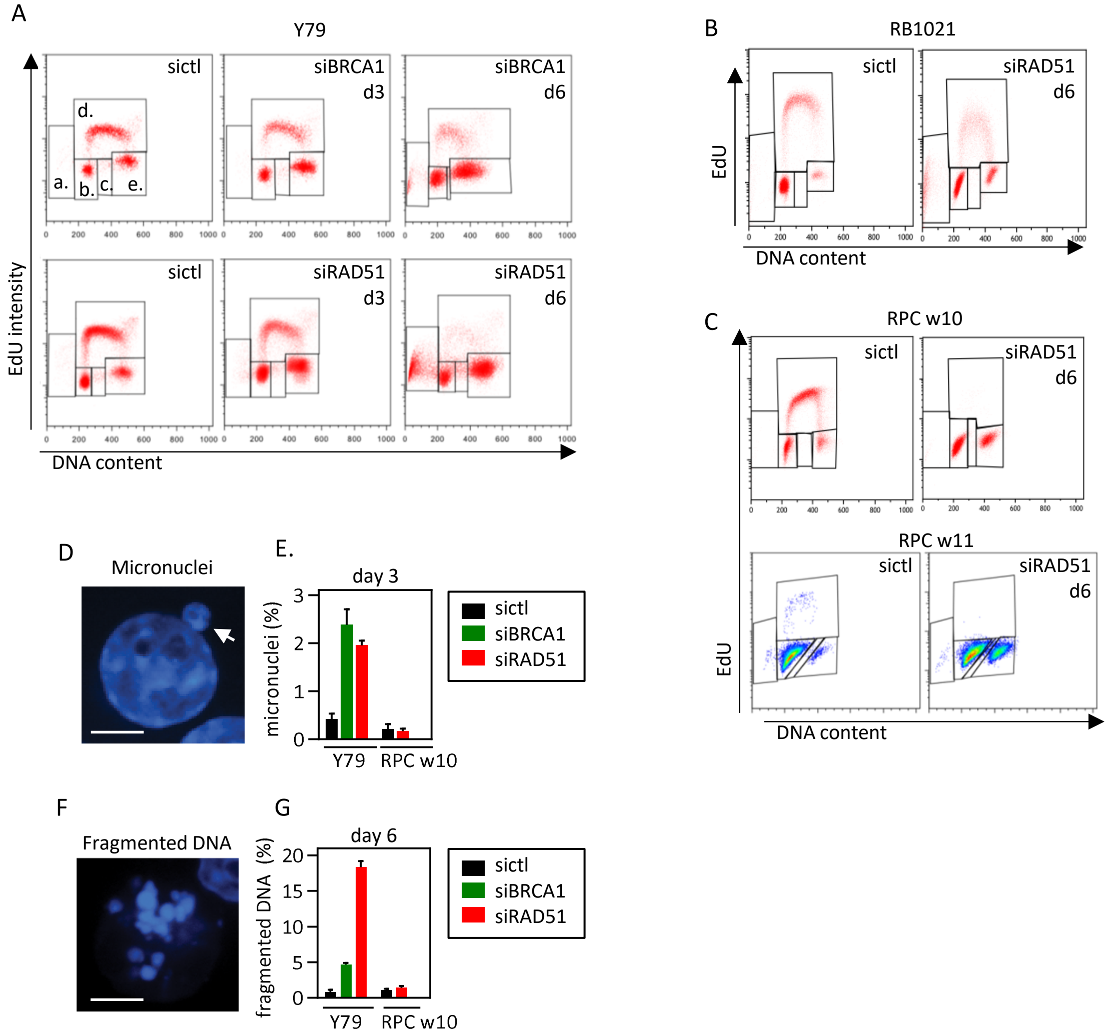

Figure S9. Effect of BRCA1 or RAD51 depletion on the cell cycle, micronuclei formation, and DNA fragmentation.

A-C Flow cytometry plots showing the cell cycle effects of BRCA1 and RAD51 depletion on the indicated RB cell lines and RPC at day 3 and/or day 6. **D-E** Micronuclei formation was assessed in Y79 and RPC treated as indicated by labeling nuclei with DAPI. A representative confocal image of a micronucleus indicated by the white arrow is shown in (D) (magnification is 120x), and quantification is shown in a bar chart in (E) (n = 2, mean +/- range). **F-G** The same approach as in (D-E) was used to detect and quantify DNA fragmentation. A representative confocal image of fragmented DNA is shown in (F) (magnification 120x), and quantification is shown in a bar chart in (G) (n = 2, mean +/- range). Scales bars are 5 µm.

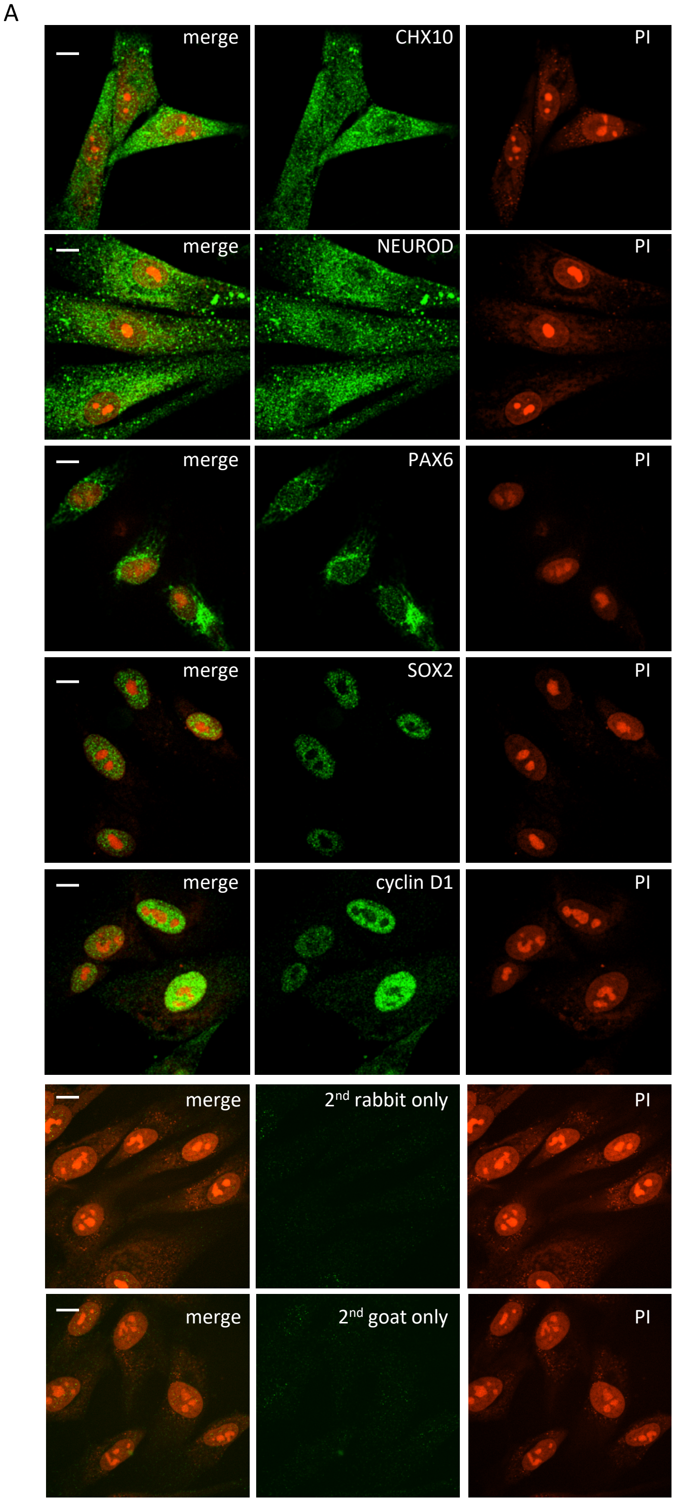

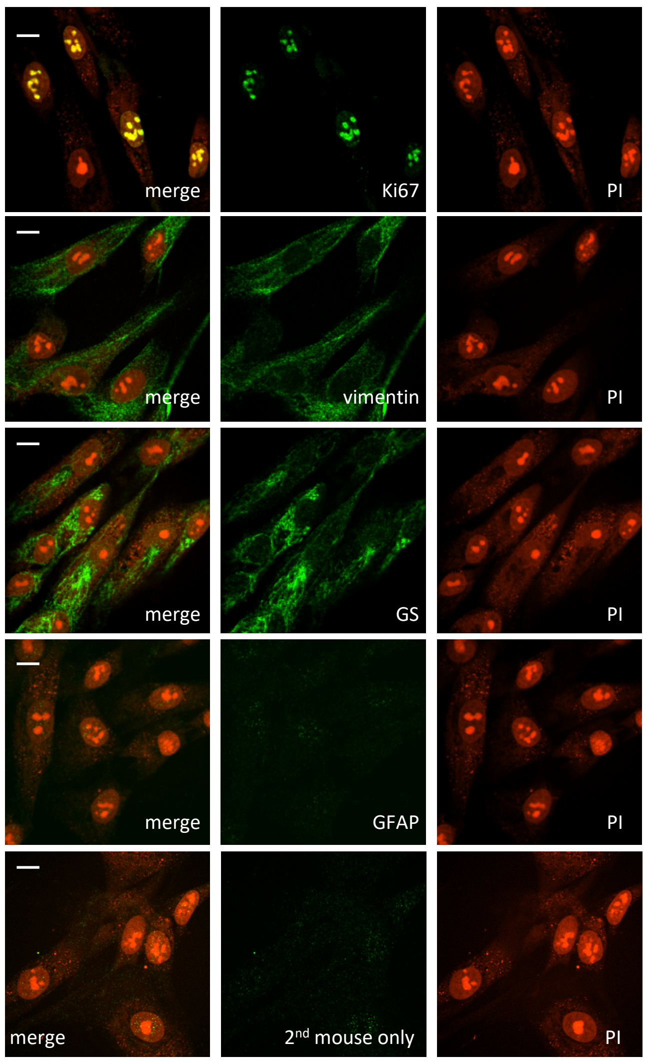

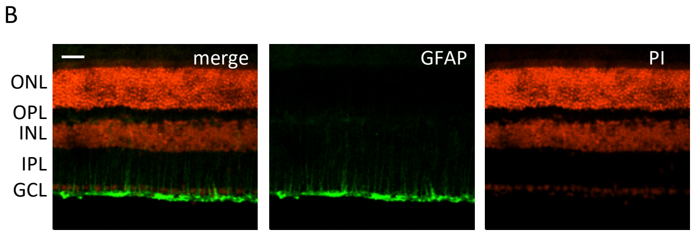

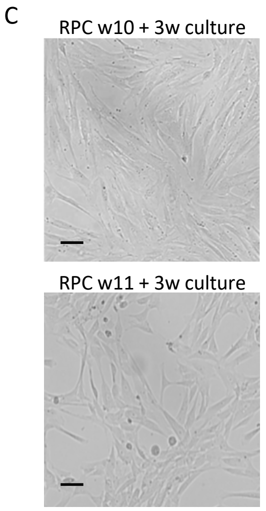

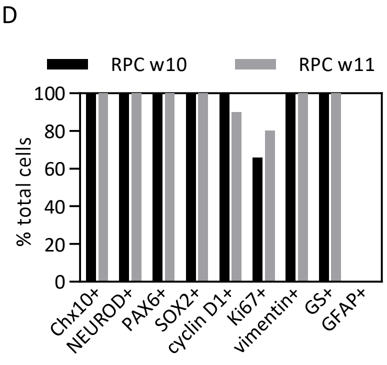

Figure S10. Retinal identity and active proliferation of dissociated RPC.

A Representative confocal images of dissociated RPC w10 immunostained with a panel of retinal markers and the proliferation marker Ki67. Negative control staining (secondary antibody only) is also shown. Magnification is 120x. **B** A murine eye section was used in parallel as positive control for GFAP immunostaining. **C** Representative bright field image of the same RPC as in (A) after 3 wk in culture. Magnification 10x. **D** Bar chart showing the proportion of cells positive for each indicated marker. At least 1000 cells from multiple random fields were quantified. Scale bars are 10 µm in A, 50 µm in B, and 20 µm in C.

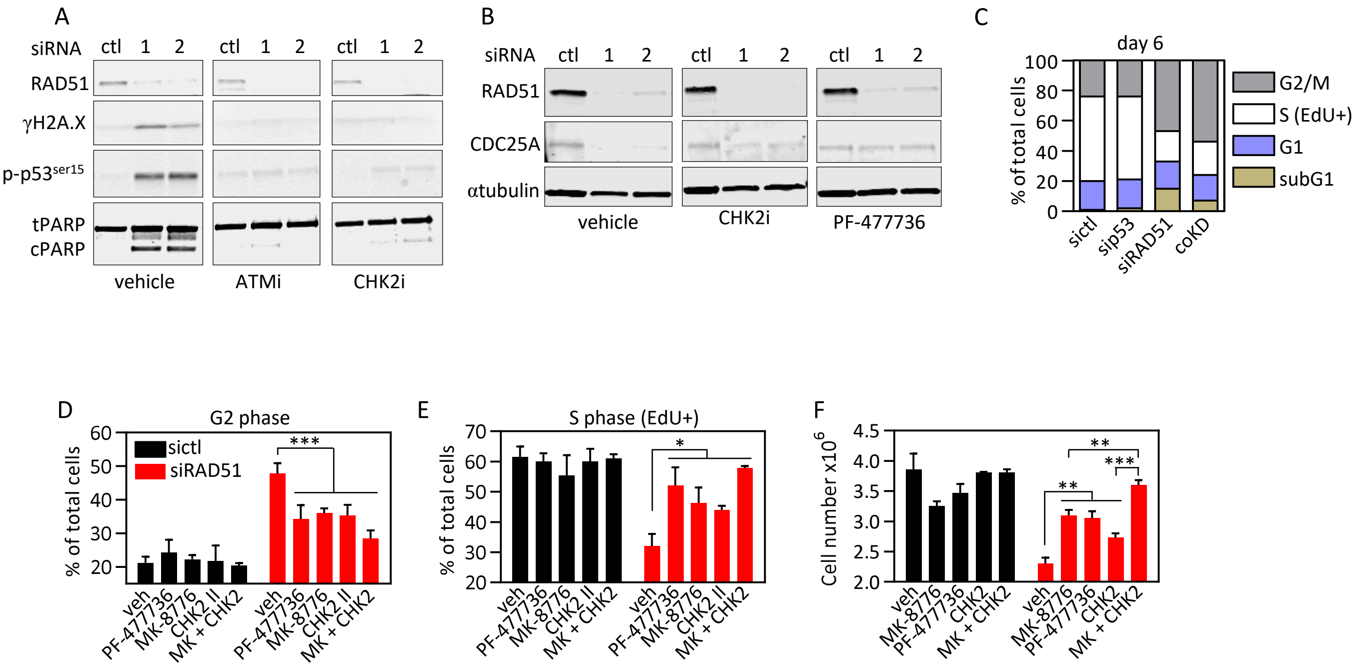

Figure S11. CHK1/2 pathways coordinate cell cycle arrest and p53-dependent death in RAD51-depleted Y79 cells.

A Y79 cells were co-treated 3 days with sictl or two different siRAD51 and vehicle, ATM or CHK2 inhibitors as indicated, then harvested for western analysis of RAD51 level, phosphorylation of H2A.X at S139 (γH2A.X) or p53 at S15, and PARP cleavage. **B** The same siRAD51 experiment as in (A) was performed in the presence of CHK1 and CHK2 inhibitors, and RAD51 and CDC25A level were assessed by western blot. **C** Y79 cells were treated with sictl or either siRAD51, sip53, or both at day 0 and 3, and at day 6 the cells were labeled with EdU/fxcycle, and assessed by flow cytometry. The proportion of cells in each cell cycle phase was normalized as percentage of total cells and plotted. **D-F** Y79 cells were treated for 3 days with sictl or siRAD51 and vehicle (veh) or the indicated CHK1/2 inhibitors alone or in combination, then stained with EdU/fxcycle. Cell cycle was analyzed by flow cytometry, data was normalized as percentage of total cells, and the proportions of cells in G2 (D) and S (EdU+; E) phases were plotted. Prior to fixation, live cells were also counted (F). n = 3, mean +/- SD, * $p < 0.05$, ** $p < 0.01$, *** $p < 0.001$ ordinary one-way ANOVA, Tukey's multiple comparisons test.

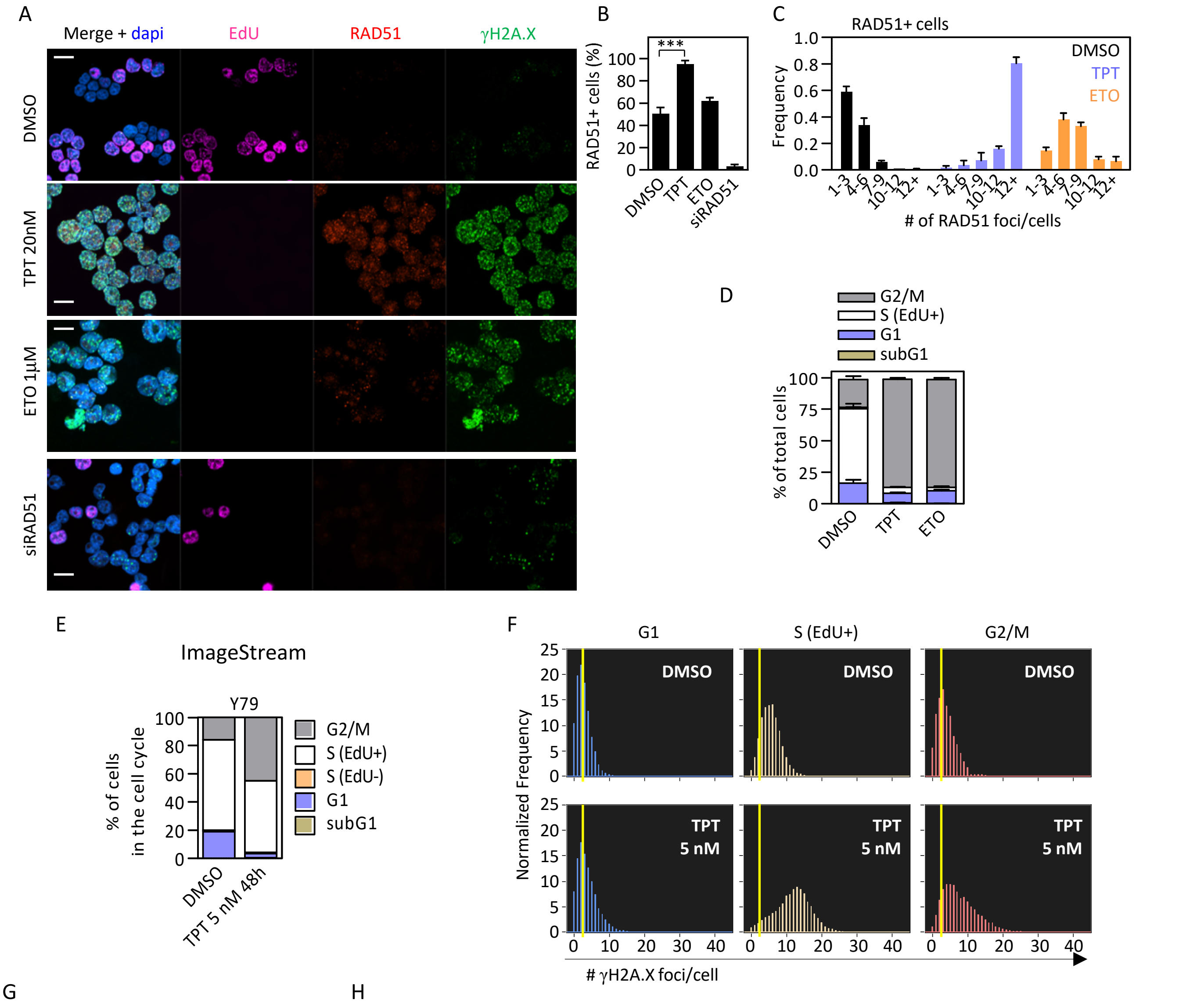

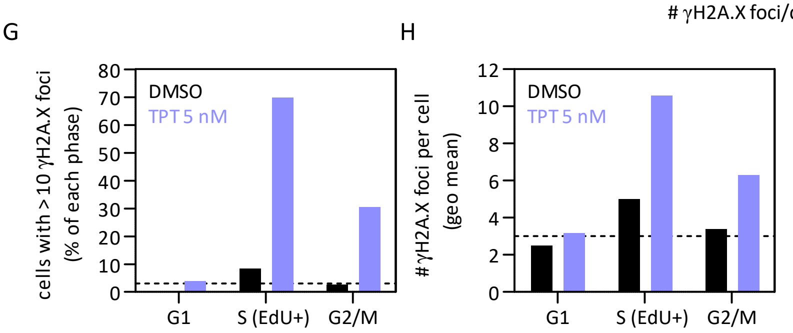

I

condition	ImageStream: cell count	ImageStream: γH2A.X + cells (% of total)	manual counting: cell count	manual counting: γH2A.X + cells (% of total)
DMSO	10199	53	278	53
TPT 5nM	23774	85	222	68

Figure S12. Topoisomerases inhibitors induce DSBs, activate RAD51, and arrest Y79 cells in G2.
A Y79 cells were treated for 3 days with Topotecan or Etoposide, then immunostained for RAD51 and γH2A.X. Foci were quantified by confocal microscopy. EdU was used to label S-phase cells. siRAD51 was used as negative control for RAD51 staining. Scale bars are 20 μm. **B** Quantification of RAD51$^+$ cells from confocal images in (**A**) (n = 3, mean +/- SD, *** p < 0.001 Student t-test). **C** Quantified distribution of RAD51 foci in RAD51$^+$ cells from confocal images in (A). **D** Y79 cells were treated as in (A) for 48h, then stained with EdU/fxcycle and cell cycle was assessed by flow cytometry. Each phase of the cell cycle was normalized and graphed as percentage of total cells (n = 2, mean +/- range). **E-H** High throughput assessment of DNA damage per cell in the cell cycle. Y79 cells were treated for 48h with 5 nM TPT, labeled with EdU/fxcycle/γH2A.X, and analyzed with the ImageStream imaging flow cytometer. Cell cycle phase distribution is shown in (E). Distribution of nuclear γH2A.X foci per cell in each phase of the cell cycle is shown in (F), where the yellow line represents the modal value in G1 cells treated with DMSO. From the graphs in (F), the proportions of cells with more than 10 γH2A.X foci were quantified and plotted as percentage of each phase (G), and the geometric means of the number of γH2A.X foci/cell were quantified and plotted (H). The dotted line in (G) and (H) corresponds to the yellow baseline in (F). **I** The same experiment as in E-H, but the proportion of γH2A.X positive cells was quantified using confocal microscopy and compared with the data obtained using ImageStream.

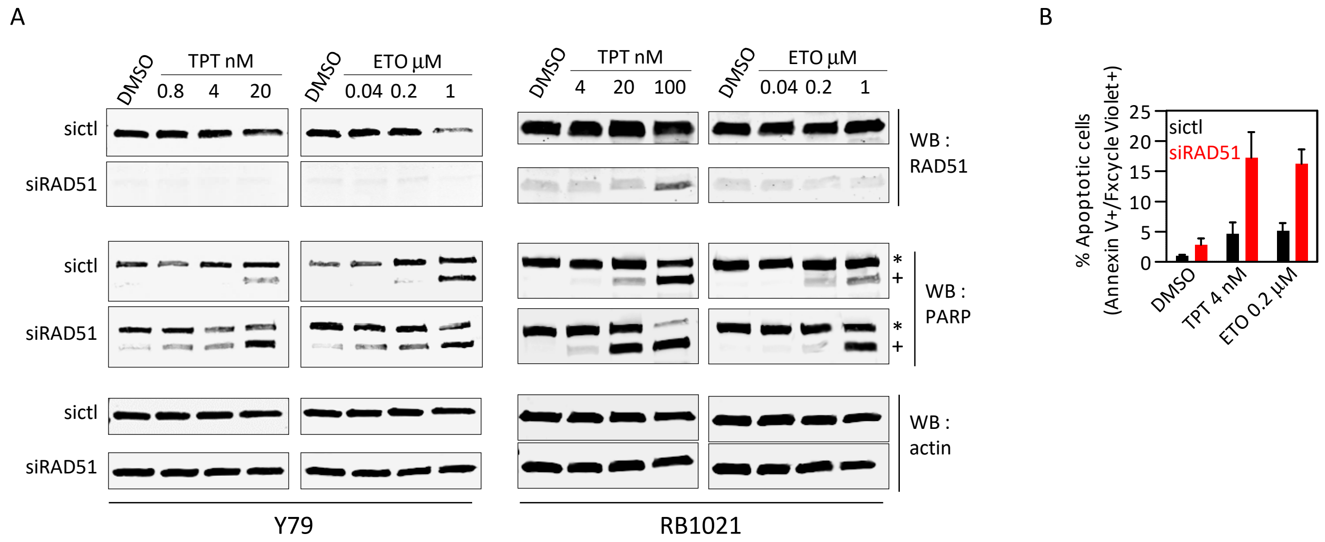

Figure S13. RAD51 knockdown enhances the apoptotic effect of topoisomerase inhibitors.
A RB cell lines were treated as indicated, and at day 3 RAD51 levels and PARP cleavage were assessed by western blot. Actin was used as loading control. Quantification of PARP cleavage is shown in **Fig. 3D**. * total PARP, + cleaved PARP**. B** Y79 were treated as indicated for 3 days and annexin V^{+};fxcycle^{+} cells quantified by flow cytometry. n = 2 +/- range.

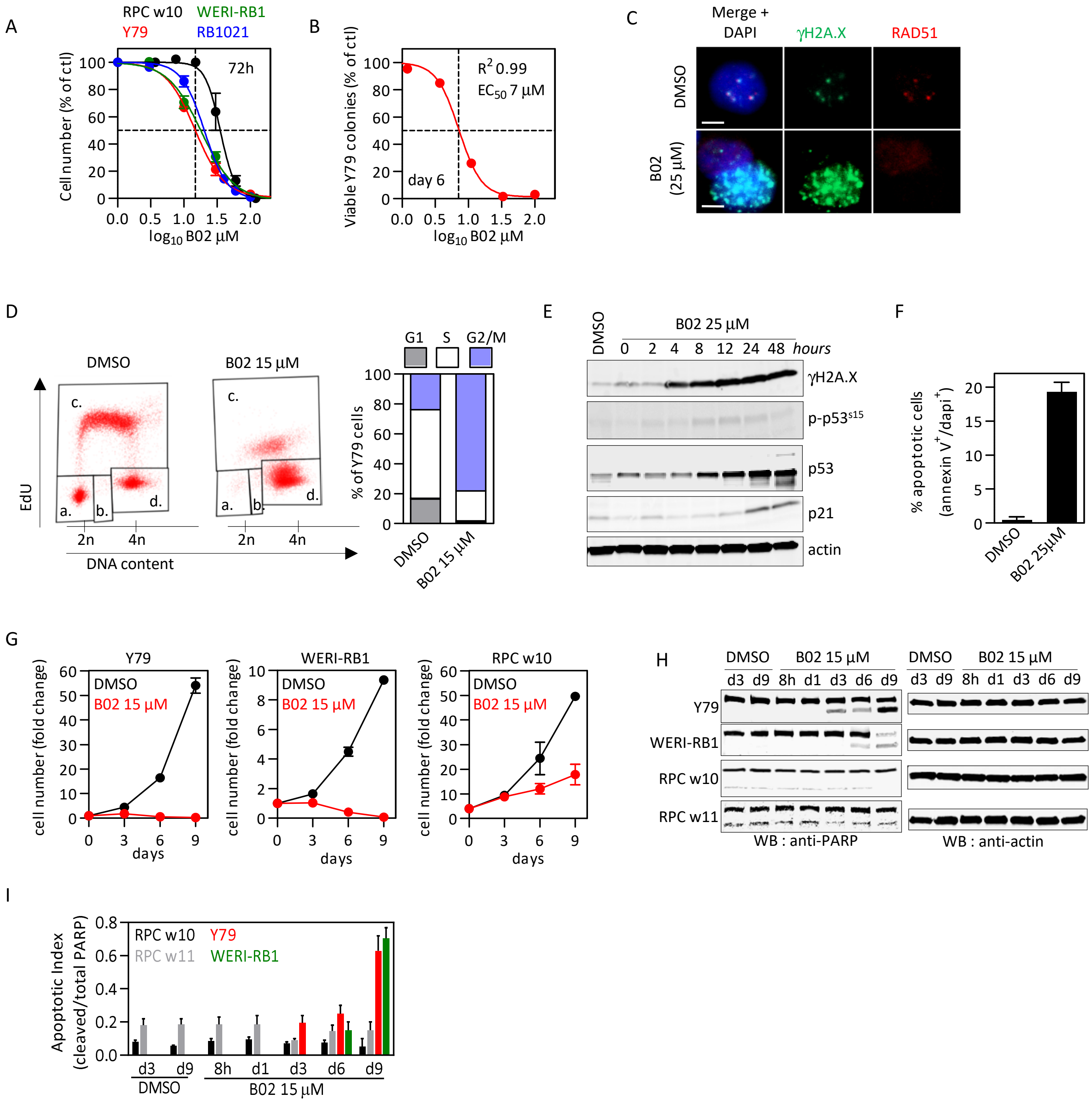

Figure S14. B02 recapitulates RAD51 knockdown.

A RB cell lines and RPC were treated with a range of concentrations of B02, and at day 3 live cells were counted with trypan blue and plotted as percentage of control (n = 2, mean +/- range). Dotted lines show 50% effect and EC50 on y and x axes, respectively. **B** Y79 cells were grown in soft agar with vehicle (DMSO) or different concentrations of B02 and at day 6 viable colonies were assessed with alamar blue. Data was plotted as percentage of DMSO and fitted. Goodness of fit (R^2) and EC50 are shown (n = 2, mean +/- range). **C** Y79 cells were treated as indicated for 2 days, immunostained for γH2A.X (green) and RAD51 (red), and analyzed by confocal microscopy. Representative images are shown. Scale bars are 5 μm. **D** Y79 cells were treated as indicated, stained at day 3 with EdU/fxcycle, and cell cycle phase proportions assessed by flow cytometry**:** a. G1; b. S (EdU-); c. S (EdU+); d. G2/M. **E** Y79 cells were treated with B02 and levels of γH2A.X, p53, p53 S15, and p21 were assessed at various exposure times by western blot. Actin was used as loading control. **F** Y79 cells were treated for 72 h with B02, then apoptotic cells were stained with annexin V/DAPI, quantified by flow cytometry, and the data graphed as percentage of total cells (n = 2, mean +/- range). **G-I** RB cell lines and RPC were treated with vehicle (DMSO) or 15 μM B02 for 9 days, and at the indicated time points live cells were counted with trypan blue, normalized to day 0 and graphed (G), and apoptosis assessed by PARP cleavage (H) and quantified (I). n = 2, mean +/- range.

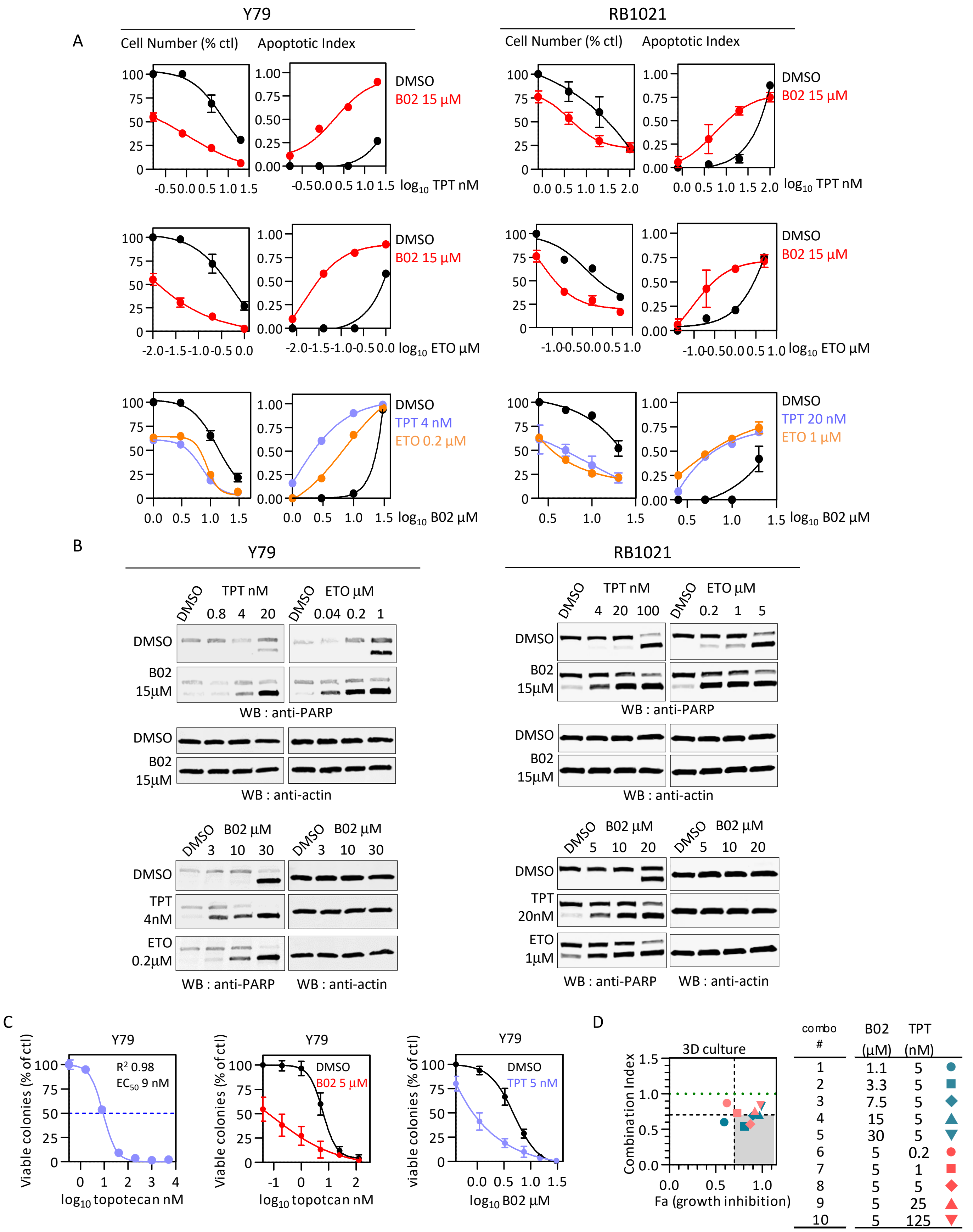

Figure S15. B02 synergy with topoisomerase inhibitors TPT and ETO.

A-B The two indicated RB cell lines were treated with different concentrations of B02, TPT, or ETO alone or in combination as indicated. At day 3, live cells were counted with trypan blue, normalized as percentage of control (A), and apoptosis was assessed in parallel by PARP cleavage (B), and quantified (A). n = 2, mean +/- range. **C** Y79 cells were grown in soft agar with vehicle (DMSO) or different concentrations of TPT, and at day 6 viable colonies were assessed with alamar blue. Data was plotted as a percentage of vehicle treated cells and fitted (n = 2, mean +/- range). Goodness of fit (R^2) and EC50 are shown. In the two graphs on the right, TPT and B02 dose responses were performed in combination with the fixed indicated concentrations of B02 and TPT, respectively (n = 2, mean +/- range). **D** CI *vs* Fa plot of the data from (C). Grey area and green line as in **Fig. 4A**.

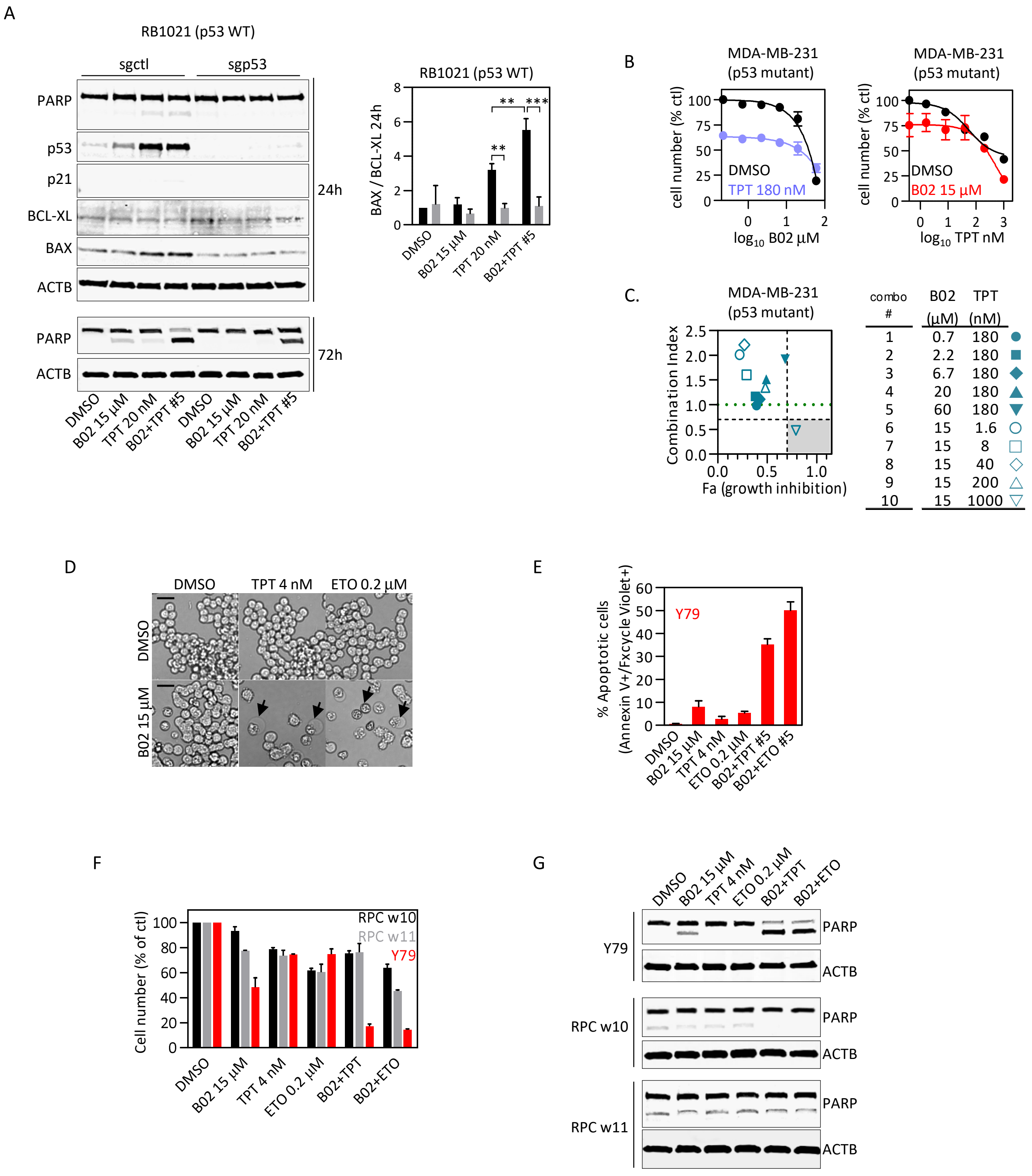

Figure S16. B02 + TPT engage p53 to synergistically kill RB cells but not RPC.
A RB1021 cells were transduced with a control or p53-targeting sgRNA lentivirus, selected in puromycin, then treated as indicated and, at 24h and 72h, westerns were run to assess p53, p21, BAX, BCL-XL levels, and PARP cleavage; the BAX/BCL-XL ratio was quantified and plotted (n = 3, mean +/- SD, ** $p < 0.01$, *** $p < 0.001$ ordinary one-way ANOVA, Tukey's multiple comparisons test). **B-C** B02+TPT synergy was assayed in the p53 mutant breast cancer line MDA-MB-231, and the 72h growth inhibition curves (B) and related CI *vs* effect (Fa) plot (C) were plotted. **D-E** The proapoptotic effect of both B02+TPT and B02+ETO combos in Y79 cells visualized under bright field microscopy (D, black arrow indicates apoptotic cells), and quantified by increased annexin V$^+$;fxcycle$^+$ cells (E; n = 2, mean +/- range). Scale bars are 20 µm. **F-G** RPC and Y79 cells were treated with the indicated single or combo drugs, and at day 3 cells were counted and plotted as percentage of control (F) (n = 2, mean +/- range), and cell lysates were run to assess PARP cleavage (G).

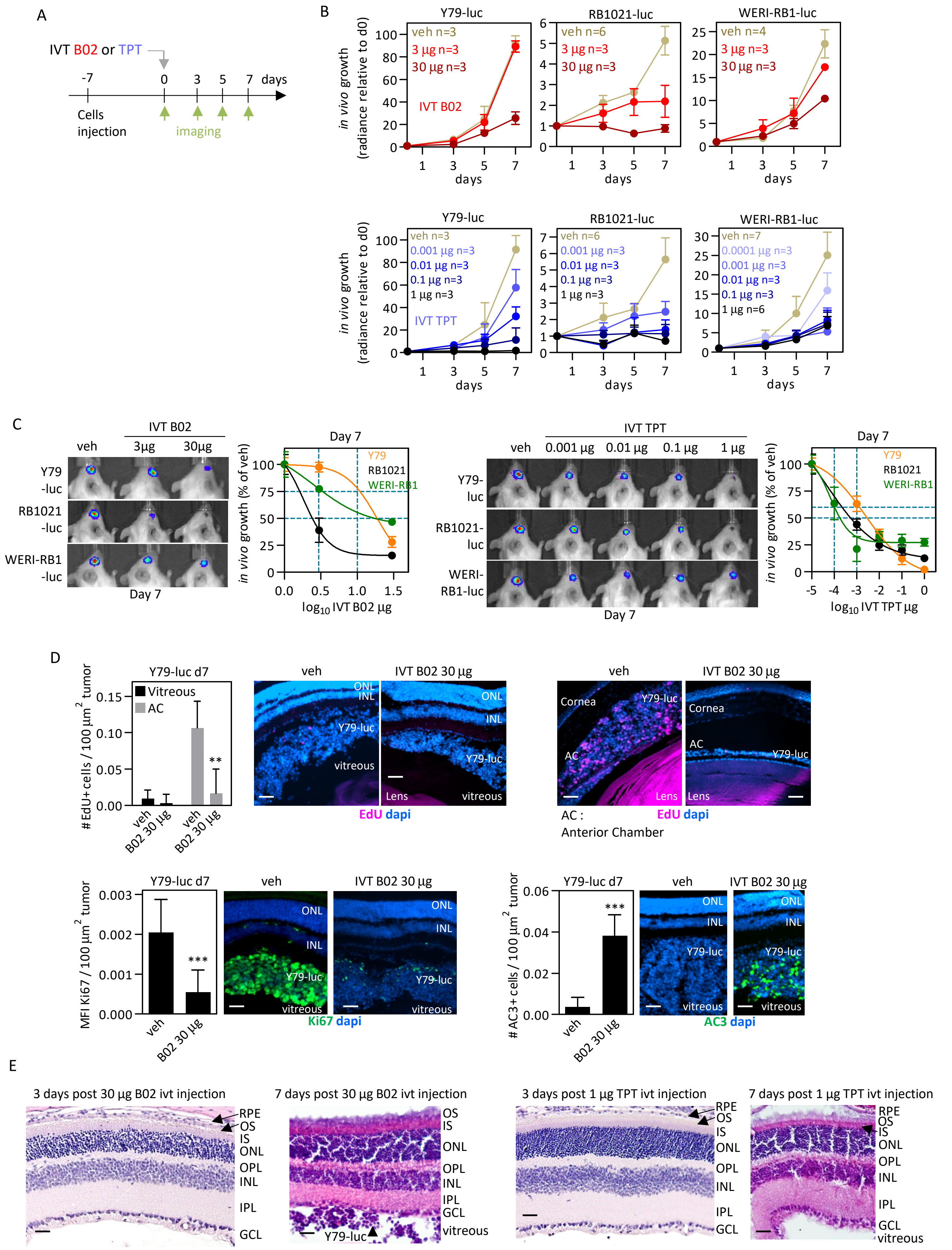
A
IVT B02 or TPT
-7 0 3 5 7 days
Cells injection
imaging
B
Y79-luc
RB1021-luc
WERI-RB1-luc
in vivo growth (radiance relative to d0)
veh n=3
3 µg n=3
30 µg n=3
IVT B02
veh n=6
veh n=4
days
0.001 µg n=3
0.01 µg n=3
0.1 µg n=3
1 µg n=3
IVT TPT
veh n=7
0.0001 µg n=3
1 µg n=6
C
IVT B02
veh 3µg 30µg
Y79 -luc
RB1021 -luc
WERI-RB1 -luc
Day 7
in vivo growth (% of veh)
Y79
RB1021
WERI-RB1
log10 IVT B02 µg
IVT TPT
veh 0.001 µg 0.01 µg 0.1 µg 1 µg
log10 IVT TPT µg
D
Y79-luc d7
Vitreous
AC
EdU+ cells / 100 µm2 tumor
veh
B02 30 µg
IVT B02 30 µg
ONL
INL
vitreous
Lens
Cornea
EdU dapi
AC : Anterior Chamber
MFI Ki67 / 100 µm2 tumor
Ki67 dapi
AC3+ cells / 100 µm2 tumor
AC3 dapi
E
3 days post 30 µg B02 ivt injection
7 days post 30 µg B02 ivt injection
3 days post 1 µg TPT ivt injection
7 days post 1 µg TPT ivt injection
RPE
OS
IS
OPL
IPL
GCL

Figure S17. B02 and TPT efficacies *in vivo* without toxicity.

A Timeline to assess IVT B02 or TPT in three orthotopic RB xenograft models. **B** *In vivo* growth curves of orthotopic RB xenografts treated with vehicle or the indicated doses of B02 or TPT. **C** The day 7 growth data of different doses of IVT B02 or TPT from (**B)** were plotted as a percentage of vehicle for each RB cell line, and fitted to delineate EC50s and subEC50s (dotted lines); representative images of the radiance signals at day 7 are shown on the left of each graph. **D** Eye sections containing Y79 xenografts (Y79-luc) treated as indicated were labeled at day 7 for active S phase (EdU, top), Ki67 (bottom left), or active caspase 3 (bottom right), then positive cells or fluorescence signal from multiple random fields of the three tumors were quantified, normalized, and data plotted as bar charts (mean +/- SD, ** $p < 0.01$, *** $p < 0.001$ Student t-test; MFI: Mean Fluorescence Intensity). For each indicated treatment, a representative confocal image of the staining is shown (ONL: Outer Nuclear Layer, INL: Inner Nuclear Layer). **E** Representative paraffin (day 3, no xenograft) and frozen (day 7, Y79 xenograft) H&E-stained murine eye sections treated with 30 µg IVT B02 or 1 µg IVT TPT. Multiple sections from three eyes for each time point were analyzed (RPE: Retinal Pigment Epithelium, OS: Outer Segment, IS: Inner Segment, ONL: Outer Nuclear Layer, OPL: Outer Plexiform Layer, INL: Inner Nuclear Layer, IPL: Inner Plexiform Layer, GCL: Ganglion Cell Layer). Cracks in the retina after 7 days are artefacts from processing of frozen sections. Scale bars are 50 µm.

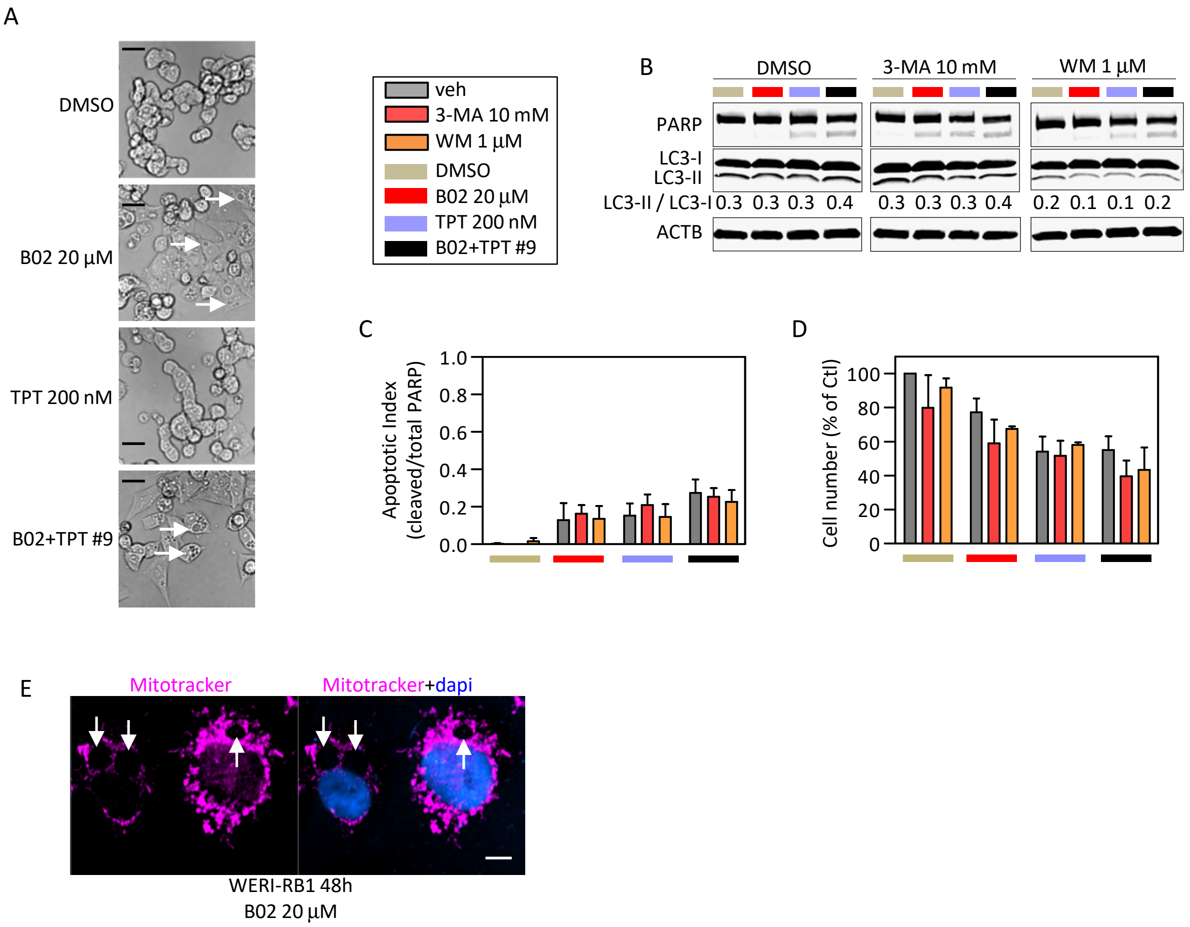

Figure S18. Autophagy-like effect of B02 is independent of resistance to B02 and TPT.
A Bright field microscopy reveals cytoplasmic vacuoles (white arrows) in WERI-RB1 cells treated with B02 or B02+TPT for 48h. Scale bar are 20 µm. **B-D** WERI-RB1 cells treated with single drugs or B02+TPT were concomitantly incubated with the indicated concentrations of the autophagy inhibitor 3-methyladenine (3-MA) or wortmannin for 3 days. Westerns were used to assess levels of the autophagy marker LC3B (ratios LC3-II/LC3-I are indicated below the relevant blot), as well as PARP cleavage (B). Extent of PARP cleavage was quantified and graphed (C). Live cell counts with trypan blue were also determined (D). n = 2, mean +/- range. **E** Mitochondria in B02-treated cells were labeled with Mitotracker, and confocal microscopy used to assess their presence or absence in cytoplasmic vacuoles. Of at least 10,000 of cells analyzed from random fields in 3 separate experiments, vacuoles (white arrows) never contained mitochondria. Scale bar is 5 µm.

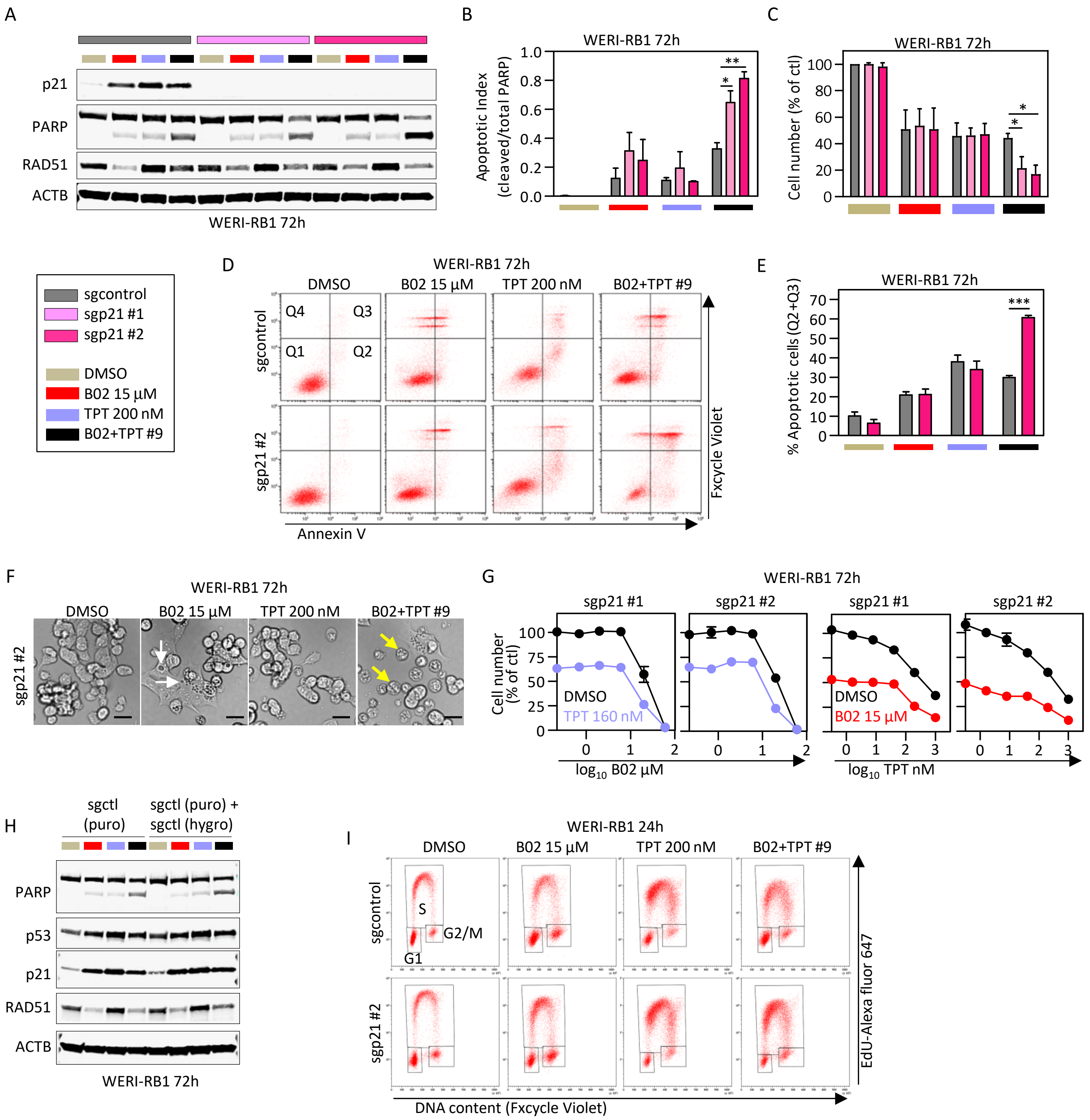

Figure S19. Deleting p21 sensitizes WERI-RB1 cells to B02+TPT.

A-F WERI-RB1 cells were transduced with a control or two different p21 sgRNAs. Sensitivity to B02, TPT, and B02+TPT (combo #9 in **Fig. 5B**: 15 µM B02, 200nM TPT) was assessed after 3 days by PARP cleavage (A, B), live cell counts (C), or annexin V^+;fxcycle$^+$ double labeling (D, E) (n = 3, mean +/- SD**,** * p < 0.05, ** p < 0.01, *** p < 0.001 ordinary one-way ANOVA, Tukey's multiple comparisons test). Bright field images show examples of vacuoles (white arrows) and apoptotic cells (yellow arrows). Scale bar are 20 µm. **G** The synergy experiment in **Fig. 5A** was repeated in the two established p21-deleted WERI-RB1 cells (n = 2, mean +/- range). The corresponding CI *vs* Fa plots are shown in **Fig. 5H**. **H** Western of WERI-RB1 cells co-transduced with control viruses, related to **Fig. 5E-G**. **I** p21-deleted cells treated for 24h as indicated were labeled with EdU and fxcycle and cell cycle phase proportions assessed by flow cytometry. Representative flow plots are illustrated, and quantification is plotted in **Fig. 5J**.
